# Redox signaling at the crossroads of human health and disease

**DOI:** 10.1002/mco2.127

**Published:** 2022-03-31

**Authors:** Jing Zuo, Zhe Zhang, Maochao Luo, Li Zhou, Edouard C. Nice, Wei Zhang, Chuang Wang, Canhua Huang

**Affiliations:** ^1^ State Key Laboratory of Biotherapy and Cancer Center West China Hospital, and West China School of Basic Medical Sciences & Forensic Medicine, Sichuan University, and Collaborative Innovation Center for Biotherapy Chengdu P. R. China; ^2^ Department of Biochemistry and Molecular Biology Monash University Clayton Victoria Australia; ^3^ West China Biomedical Big Data Center West China Hospital Sichuan University Chengdu P. R. China; ^4^ Mental Health Center and Psychiatric Laboratory The State Key Laboratory of Biotherapy West China Hospital of Sichuan University Chengdu P. R. China; ^5^ Department of Pharmacology Provincial Key Laboratory of Pathophysiology, Ningbo University School of Medicine Ningbo Zhejiang P. R. China

**Keywords:** hydrogen peroxide, oxidative stress, reactive oxygen species, redox‐relevant disease, redox signaling, redox therapy

## Abstract

Redox biology is at the core of life sciences, accompanied by the close correlation of redox processes with biological activities. Redox homeostasis is a prerequisite for human health, in which the physiological levels of nonradical reactive oxygen species (ROS) function as the primary second messengers to modulate physiological redox signaling by orchestrating multiple redox sensors. However, excessive ROS accumulation, termed oxidative stress (OS), leads to biomolecule damage and subsequent occurrence of various diseases such as type 2 diabetes, atherosclerosis, and cancer. Herein, starting with the evolution of redox biology, we reveal the roles of ROS as multifaceted physiological modulators to mediate redox signaling and sustain redox homeostasis. In addition, we also emphasize the detailed OS mechanisms involved in the initiation and development of several important diseases. ROS as a double‐edged sword in disease progression suggest two different therapeutic strategies to treat redox‐relevant diseases, in which targeting ROS sources and redox‐related effectors to manipulate redox homeostasis will largely promote precision medicine. Therefore, a comprehensive understanding of the redox signaling networks under physiological and pathological conditions will facilitate the development of redox medicine and benefit patients with redox‐relevant diseases.

## Graphical abstract

Redox homeostasis is essential for human health, supported by multiple functional signaling pathways including the Keap1‐Nrf2, FOXO, HIF and NF‐κB pathways. However, redox imbalance causes multiple serious diseases such as type 2 diabetes, atherosclerosis, chronic obstructive pulmonary disease, Alzheimer's disease, cancer, and aging. Our review emphasizes the significance of redox manipulation in clinical therapeutics and points out the existing challenges involved in this field.

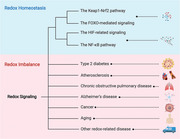

## 1 INTRODUCTION

Electron flow is one of the most fundamental and common perspectives in understanding biology.^1^ As Nobel prize‐winning biochemist Albert Szent‐Györgyi said, “Life is nothing but an electron looking for a place to rest.” Although life seems to be much more complex than electrons, the electron transfer process, which refers to the transformation of energy from an excited state (high‐energy electrons) to a ground state (low‐energy resting electrons) is fundamental. The rules of quantum mechanics restrain the electron transfer process whereby high‐energy electrons drop spontaneously to low‐energy states. Instead, they need to be offered feasible pathways, which tightly regulate the rearrangement of electrons along the energy scale. These electron transfer and transformation of energy processes make life possible.^1^

The delivery of electrons from the donor molecules to the terminal acceptor molecules triggers oxidative (for donor molecules) and reductive (for acceptor molecules) processes, defined as redox reactions. Redox reactions are the sources of intracellular energy, harvested from electron flow during the transfer from one reductant (oxidation) to another oxidant (reduction). High‐energy electrons must move to a new resting acceptor, usually molecular oxygen for aerobes, ultimately yielding H_2_O. For humans, intracellular enzymes reduce oxygen, which enters the cell and captures energy to accelerate ATP production. However, in some specific conditions (i.e., redox control), the reduction of oxygen is blocked at an intermediate state, such as hydrogen peroxide (H_2_O_2_), which functions as an active oxidant to modulate multiple physiological and pathological redox signaling pathways on the basis of the level of oxidants.^2^, ^3^

The delivery of electrons on the basis of energy gradients prompts the rearrangements of chemical bonds and induces cellular responses at all levels of regulation, triggering redox chemistry and redox biology simultaneously. However, how does electron flow arouse resting cells and instruct them to determine cell fate, from proliferation and differentiation to death? Many essential intermediate proteins are involved between electron flow and cellular responses, whose activities are dynamically modulated by the electron transfer process through disulfide bridges. These functional proteins act as the main executors in regulating cellular biological processes and making cell fate decisions. In the broadest sense, identifying the coordination and crosstalk between electron gradients and cellular responses is the main purpose of redox biology. Nevertheless, understanding the redox modification of these functional intermediate proteins seems to be equally important due to the close correlation of multiple diseases with aberrant redox modulation, which will largely promote the development of redox medicine. Hence, redox reactions are intimately linked to human health, in which the concept of redox homeostasis is emphasized. Constant intracellular surveillance is vital for maintaining redox homeostasis, termed “homeodynamics” due to its dynamic property.^4^

Recent studies on redox biology have indicated a relatively complete redox architecture that is closely linked to physiological function,^5^ with a set of intricate mechanisms denoted as the “redox code.”^6^ H_2_O_2_ as a key second messenger is central to the redox code, contributing to cell fate decisions.^6^ In addition, the redox modifications of cysteines, such as intra‐ or intermolecular disulfide bond formation, *S*‐sulfonation, *S*‐glutathionylation, and *S*‐nitrosylation, have also been reported to reset the function of proteins, thus, mediating the process of various cellular biological events.

Redox imbalance between oxidants and antioxidants, especially for oxidative stress (OS), accounts for many diseases including neurological disorders, immune system disorders, cardiovascular diseases, and skeletal diseases.^7^ Nonetheless, not all redox‐related disorders are caused by excessive reactive oxygen species (ROS) production. Several pathological conditions may also result from other reactive species, such as reactive nitrogen species and reactive sulfur species, or other small signaling molecules, such as H_2_, NH_3_, and CO, which are also capable of engendering redox imbalance.

In this review, we look back at the evolution of redox biology and summarize the cellular redox landscape with a particular focus on the core redox signaling metabolite–H_2_O_2_.^2^, ^8^, ^9^ Considering the central role redox plays in determining cell fates and overall organization of living organisms, we also discuss the impacts of redox imbalance on several human diseases with an emphasis on the enormous potential of developing redox medicine. It is hoped that this systematic perspective will facilitate a better understanding of redox biology and attract more interest in the field.

## 2 AN OVERVIEW OF REDOX BIOLOGY FROM A HISTORICAL PERSPECTIVE

Oxygen originates from the photosynthetic activity of cyanobacteria which first entered the earth's atmosphere approximately 2.3 billion years ago.^10^, ^11^ The dramatic increase of O_2_ killed most anaerobic living creatures present at that period, with only a few aerobic life forms emerging that gradually occupied a major part in all organisms. The existence of enough O_2_ and its significance in producing energy for aerobes prompted the coining of the term “redox biology.”

**FIGURE 1:**
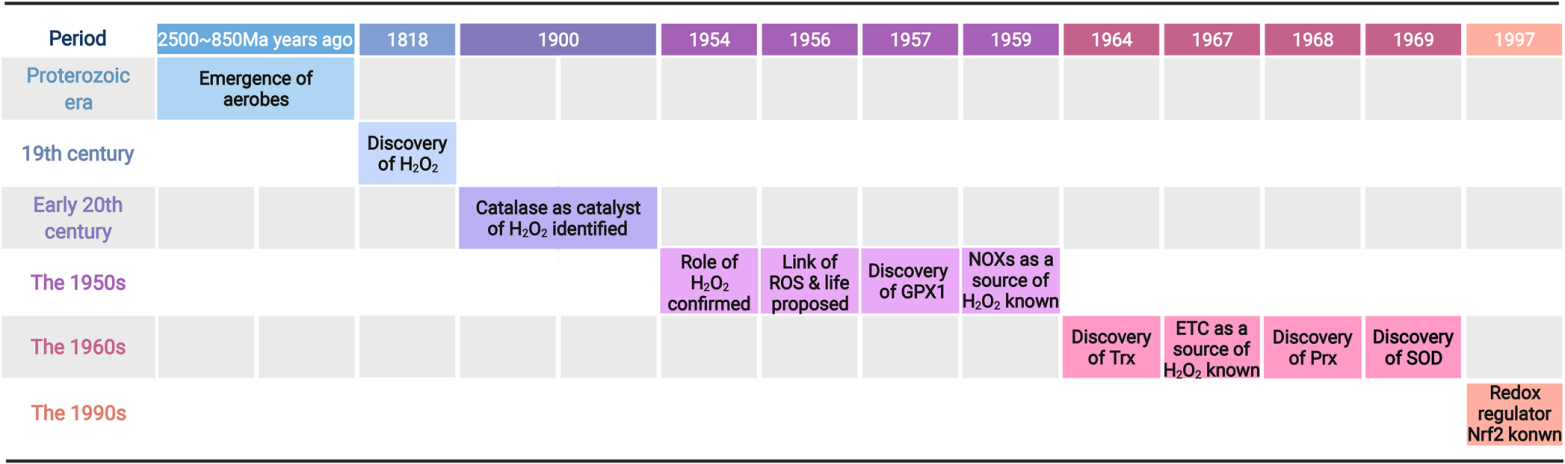
Key events in the history of redox biology

Flohe and de Villiers et al. have reviewed several important events at the early stages of redox biology,^11^, ^12^ which we have addressed and added other representative findings along this timeline (Figure 1). Generally, oxygen is highly active and can be transformed into multiple ROS, such as H_2_O_2_, singlet molecular oxygen(1O_2_), ozone (O_3_), superoxide anion radicals (O_2_^−•^), and hydroxyl radicals (OH**^.^**), by endogenous and exogenous factors.^13^, ^14^ Among the major members of ROS family, H_2_O_2_ is the most frequently explored and first discovered in redox chemistry by Louis Jaques Thénard in 1818,^12^ though the underlying roles in redox biology were not defined until 1954.^15^ In 1900, catalase, which may be the first discovered antioxidant, was defined as a catalyst of H_2_O_2_.^16^ Intriguingly, selenium was first found and identified as a toxic chemical catalyst in 1818 and was later proven to be an essential component of the glutathione peroxidase (GPX) family.^12^ GPX was claimed to be a novel peroxidase that was independent of typical heme peroxidase in 1957, which was initially widely debated, but has gradually been accepted as GPX1 in the GPX family.^17^ In addition to the GPX family, thioredoxin reductases (TrxR), as catalysts of Trx, are well‐studied selenoproteins that are classified as important oxidoreductases. Trx and peroxiredoxin (Prx) were first discovered in 1964 and 1968, respectively.^18^, ^19^ They function as two essential antioxidant systems that determine cell fates and pathophysiological changes in response to various stresses.^20^, ^21^, ^22^, ^23^, ^24^, ^25^

Intracellular H_2_O_2_ is endogenously derived from NADPH oxidase (NOX), mitochondrial oxidative phosphorylation (mtOXPHOS ) (i.e., the electron transport chain [ETC] of mitochondria), and other cell compartments, including peroxisomes and the endoplasmic reticulum (ER),^2^, ^26^ or exogenously arises from stress such as ultraviolet radiation, ionizing radiation, and toxic compounds. Early in 1961, Iyer et al. found that H_2_O_2_ was released from phagocytosis of guinea pig polymorphonuclear leukocytes.^27^ Later, in 1964, Rossi and colleagues confirmed that NOX was the upstream event in H_2_O_2_ production, answering how phagocytes generated H_2_O_2_ by respiratory burst.^28^ However, NOX was first disclosed to generate superoxide radicals and H_2_O_2_ by Sbarra and Karnowski in 1959.^29^ In 1973, a study by Babior et al. confirmed that in the process of respiratory burst, O_2_^−•^ but not H_2_O_2_ was the direct product, meaning that H_2_O_2_ originated from O_2_^−•^ during metabolic activity.^30^ In fact, before this finding, in 1969 McCord and Fridovich reported that O_2_^−•^ can be converted into two new forms–oxygen and H_2_O_2_ by bovine erythrocyte‐derived superoxide dismutase (SOD) ^31^. ETC of mitochondria was first proven to be the source of H_2_O_2_ in 1967,^32^ and in 1974, Loschen et al. similarly elucidated that O_2_^−•^ was also the precursor of H_2_O_2_ in mitochondria.^33^

For a long time, ROS were identified as toxic byproducts of redox reactions, damaging biomacromolecules (e.g., DNA,^34^, ^35^, ^36^ RNA,^37^, ^38^ proteins,^39^ and lipids^40^, ^41^, ^42^) and causing cell death or malignant transformation. In 1954 and 1956, Gershman and Harman described that oxidant burden was closely correlated with tissue injury and aging, which greatly promoted the theory of ROS as poisonous byproducts.^43^, ^44^ However, when in‐depth studies were carried out, the roles of ROS in not only damaging but also regulating physiological signaling pathways depending on ROS levels were defined.^2^, ^7^ Thus, for H_2_O_2_, under the tight control of cellular physiological signaling and antioxidant systems, the concentration of intracellular H_2_O_2_ is maintained at a physiological level (ranging from 1–100 nM) and modulates cell proliferation, differentiation and death, whereas a supraphysiological H_2_O_2_ level (above 100 nM) destroys biomacromolecules that control cell fates. In between these levels, there is a small window of the adjustable interval, where intracellular adaptive antioxidant systems are activated through Kelch‐like ECH‐associated protein 1‐NF‐E2‐related factor 2 (Nrf2/Keap1) and nuclear factor kappa B (NF‐κB), which partially reverse the detrimental impacts. Nrf2, which can induce the expression of phase II detoxifying enzyme genes through antioxidant response elements (AREs), was defined as a master regulator of antioxidant systems in 1997.^45^ A typical example of ROS acting as an essential physiological signaling agent is reflected in immune defense,^13^ in which intracellular H_2_O_2_ triggers the migration of leukocytes to lesions through several possible mechanisms.^46^, ^47^, ^48^, ^49^

In summary, research in the last century has found multiple pivotal redox regulators and disclosed their roles in redox modulation. In addition, the main sources of ROS were also partially illustrated. Additionally, ROS was found to have more functions including physiological and pathological significance. Currently, the role of redox biology is continually being extended. For instance, researchers have found more NOX subunits in recent years.^50^, ^51^

## 3 REDOX SIGNALING NETWORKS IN CELLULAR HOMEOSTASIS

### 3.1 H_2_O_2_ as the main messenger in redox signaling

**FIGURE 2:**
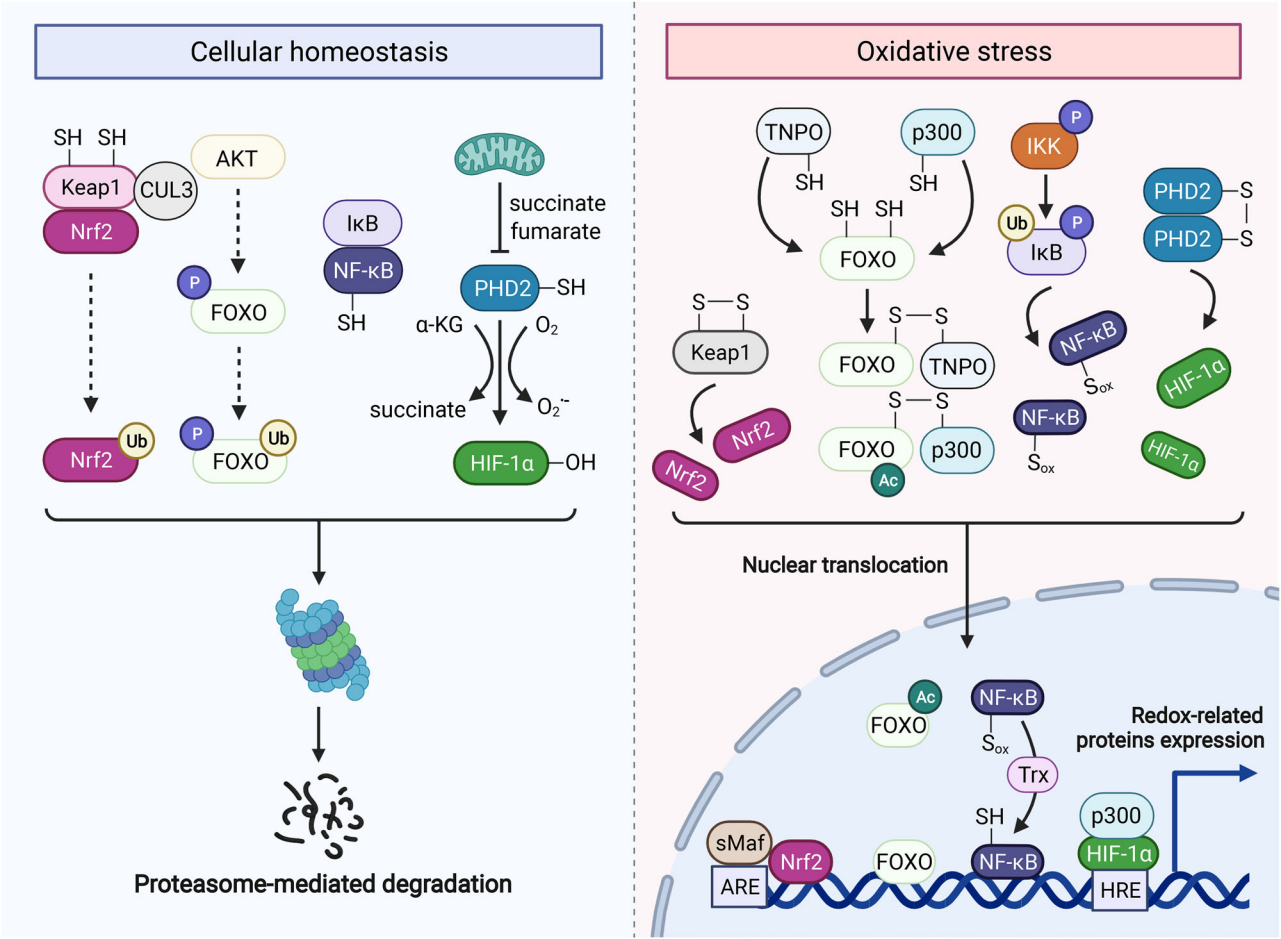
Redox regulation of Keap1‐Nrf2, FOXO, NF‐κB, and HIF. Keap1‐Nrf2: Keap1 binds to Nrf2 and CUL3, inducing the proteasomal degradation of Nrf2. Keap1 as a redox sensor is tightly regulated by H_2_O_2_, which helps to form an intramolecular disulfide and releases NRF2. FOXO: Under moderate levels of H_2_O_2_, FOXO engages proteasomal degradation through AKT signaling. However, pathological levels of H_2_O_2_ facilitate the formation of an intermolecular disulfide between TNPO and FOXO, promoting the nuclear translocation of FOXO. Intriguingly, FOXO can also form an intermolecular disulfide with acetyltransferase p300, enhancing the activity of FOXO. NF‐κB: Normally, NF‐κB remains inactive in the cytoplasm by interacting with IκB that can be phosphorylated and inhibited by IKK. Under OS, H_2_O_2_‐mediated IKK activation drives the proteasomal degradation of IκB and subsequent nuclear translocation of NF‐κB. Notably, NF‐κB is also oxidized by H_2_O_2_, which needs to be reversed by Trx in the nucleus. HIF: The redox sensor PHD2 inactivates HIF‐1α via hydroxylation, which can be prevented by forming intermolecular disulfide between PHD2 under H_2_O_2_ regulation. As a result, HIF‐1α enters the nucleus and prompts the transcription of target genes

The most physiologically relevant ROS include O_2_^−•^ and H_2_O_2_, which are generally derived from NOX and ETC. Cellular compartmentalization of H_2_O_2_ facilitates new levels of regulation and confines certain signaling pathways to specific compartments.^52^ Therefore, H_2_O_2_ is considered as one of the main types of ROS involved in redox regulation of intracellular biological processes,^2^, ^53^ the generation of which is tightly controlled through various growth factors, chemokines, and physical stress.^26^ Intracellular physiological levels of H_2_O_2_ (1–100 nM) act as major activators to modulate specific targets through reversible oxidative modification and further influence the activity, localization, and interactions of protein targets and cellular behaviors in response to moderate environmental stresses. The regulation of biological activities under physiological levels of H_2_O_2_ is also called “oxidative eustress.” However, supraphysiological intracellular H_2_O_2_ levels (above 100 nM) cause cell and tissue dysfunction, attributed to indiscriminate attacks on biomacromolecules. Excessive and irreversible oxidation directly leads to cell senescence, death, and even malignant transformation (“oxidative distress” compared to “oxidative eustress”), a term defining redox imbalance in favor of oxidant burden. In the next section, we mainly focus on H_2_O_2_ functioning as a physiological modulator in the redox regulation of four transcription factors (TFs)—Keap1‐Nrf2, forkhead box class O (FOXO), hypoxia‐inducible factors (HIFs), and NF‐κB (Figure 2).

### 3.2 ROS scavenging: The Keap1‐Nrf2 pathway

The Keap1‐Nrf2 pathway is a thiol‐based redox switch that responds to various oxidant stresses and, thus, plays an essential role in maintaining redox homeostasis in eukaryotes.^54^, ^55^ Keap1 functions as a redox sensor, inhibiting Nrf2 activity by targeting Nrf2 for ubiquitination and consequent degradation under quiescent circumstances. But under oxidative insults, Nrf2 is released from Keap1 and acts as a master TF to induce the expression of proteins involved in the cellular antioxidant responses.^56^, ^57^, ^58^

Nrf2, along with Nrf1, Nrf3, and p45 NF‐E2, is a member of the Cap “n” Collar family.^59^, ^60^ There are seven functional domains (Neh1‐7) that regulate the stability and transcriptional activity of Nrf2.^61^ The *N*‐terminal Neh2 domain is involved in the Keap1‐Nrf2 interaction,^62^ as well as Nrf2 stability and ubiquitination.^63^ The Neh4 and Neh5 domains are transcription activation domains that interact with the CREB binding protein (CBP) to promote Nrf2 transcription,^64^ leading to enhanced expression of Nrf2‐targeted ARE genes. It has been reported that retinoid X receptor alpha (RXR‐α) suppresses the function of Nrf2 by interacting with the Neh7 domain (also known as the RXR‐α interaction domain).^65^ The Neh6 domain regulates Nrf2 degradation in the absence of Keap1.^66^ The Neh1 domain enables Nrf2 to connect to the ARE sequence and activate transcription through its basic leucine zipper motif.^67^ Additionally, the Neh1 domain is important for the stability of the Nrf2 protein by interacting with the ubiquitin‐conjugating enzyme UbcM2.^68^ Following its release from Keap1, the Neh1 domain mediates the nuclear translocation of Nrf2.^69^ The C‐terminal Neh3 domain is required for interaction with the chromo‐ATPase/helicase DNA‐binding protein family member CHD6,^70^ which is required for the transcription of ARE‐dependent genes.

Keap1, a repressor of Nrf2, is composed of four domains: broad complex‐Tramtrack‐Bric‐a‐brac (BTB), intervening region (IVR), double‐glycine repeat (DGR; also known as Kelch domain), and C‐terminal region (CTR).^71^ The BTB domain has been reported to be associated with dimer formation.^72^ The IVR domain contains a nuclear export sequence (NES), which is important for the cytoplasmic localization and reactivity of Keap1 in response to oxidative stimuli. This is mediated by two reactive cysteine residues in the IVR: cysteine 273 (C273) and cysteine 288 (C288).^71^, ^73^ The two DGR/Kelch domains recruit Neh2 by differentially binding to the ETGE and DLG motifs of the Nrf2 molecule.^74^, ^75^ When oxidants are present, the DLG motif is freed from Keap1, preventing Nrf2 ubiquitination and subsequent destruction.^76^, ^77^ The interaction between Nrf2 and the DGR domain in Keap1 is competitively inhibited by proteins with specific motifs such as p62 and partner and localizer of BRCA2 (PALB2).^78^ The CTR of Keap1 has also been reported to be essential for interacting with Nrf2 and subsequent suppression.^79^, ^80^

Functioned as a physiological thiol‐based sensor–effector mechanism, the Keap1‐Nrf2 system plays a central role in responding to oxidant stress and maintaining cellular redox homeostasis.^7^, ^81^ Normally, Nrf2 binds to the E3 ligase Keap1, continually targeting Nrf2 for proteasomal destruction through Cul3‐mediated ubiquitination.^82^, ^83^ Nrf2 disassociates from the Keap1‐Nrf2 complex and translocates to the nucleus under OS circumstances. More than 500 antioxidant genes involved in redox balance and stress response are induced by Nrf2.^84^, ^85^ Keap1 harbors several cysteine sensors that can sense Nrf2‐activating chemicals, with oxidation of critical cysteine residues and induction of conformational changes in Keap1.^86^, ^87^ As a result, the Nrf2‐Keap1 interaction is partially disrupted, preventing Nrf2 ubiquitination and subsequent destruction, as mentioned above. However, a study has also shown that, in some cases, Nrf2 may not be released from Keap1.^88^ Additionally, it was found that de novo synthesis of Nrf2 occurs extremely rapid in response to low concentrations of H_2_O_2_ at a pace that surpasses the rate of Nrf2 nuclear translocation.^88^ This may be another indication that cells may have other powerful, but as yet unidentified, redox sensors associated with Nrf2 activation in addition to Keap1.

### 3.3 ROS scavenging: The FOXOs‐mediated signaling

FOXO TFs are critical regulators of the physiological stress response and have been reported to be activated in response to OS.^89^, ^90^, ^91^ The term “fork head” was first described in *Drosophila* as a potential transcriptional regulator,^92^ and it was discovered to contain a so‐called winged‐helix DNA binding domain that exists in other transcriptional regulators.^93^, ^94^, ^95^ Members of the FOXO family (or FOXO orthologues) have been found in a variety of species. For instance, FOXO is also known as daf‐16 in worms^96^ and dFOXO in flies.^97^ FOXO1a, FOXO3a, FOXO4, and FOXO6 are the four FOXO proteins found in humans and are widely expressed in a range of organs.^91^, ^98^ FOXOs have a conserved nuclear localization signal domain, a NES domain, a DNA‐binding domain, and a C‐terminal transactivation domain (TAD) that regulate their transactivation activities.^99^ The activity of FOXO proteins is mainly controlled by post‐transcriptional modifications and cytoplasmic or nuclear distribution. AKT functions as a primary regulator of FOXO phosphorylation through IKK and IκB kinases. The phosphorylated FOXOs bind to the 14‐3‐3 protein, resulting in the ubiquitination and degradation of FOXO proteins in the cytoplasm.^100^ Under OS, arginine methyltransferase 1 (PRMT1) can bind to and methylate FOXOs, which blocks AKT‐mediated phosphorylation of FOXOs, and promotes subsequent nuclear translocation.^101^

Various cysteine residues have been identified in FOXO proteins: FOXO1 has seven cysteine residues,^102^ FOXO3a and FOXO4 have five,^103^ and FOXO6 has ten cysteine residues.^89^ Oxidative modifications on these cysteines and consequent contribution to redox signaling play crucial roles in the regulation of OS.^104^, ^105^, ^106^ The oxidation of cysteines in FOXOs participates in stabilizing protein–protein interactions by promoting the formation of disulfide bridges with transportin (TNPO) in an oxidative state, which is required for the activation of FOXOs.^107^, ^108^ Furthermore, the FOXO target genes that encode antioxidant proteins are involved in suppressing the production of oxygen. For instance, Mn‐SOD (SOD2) is a FOXO‐regulated antioxidant that catalyzes the dismutation of superoxide to oxygen and H_2_O_2_^109^. H_2_O_2_ is further dismutated into water and oxygen by catalase, which is regulated by FOXO3a.^110^ In addition, FOXO3a has been demonstrated to alter the expression of mitochondrial TrxR2 and Trx2, which might contribute to the reduction of mitochondrial Prx3.^111^ Taken together, this evidence demonstrates that FOXOs function as regulators in response to OS and maintain redox homeostasis.

### 3.4 ROS generation: The hypoxia‐inducible factor‐related signaling

HIF is a TF that binds to particular nuclear cofactors and transactivates a wide range of genes in response to low oxygen levels.^112^, ^113^ HIF is a heterodimer made up of two subunits: an oxygen‐labile subunit (HIF‐α, including HIF‐1α, HIF‐2α, and HIF‐3α) and a common stable β‐subunit (HIF‐1β/ARNT).^114^, ^115^ Both subunits are members of the basic helix‐loop‐helix (bHLH‐PAS) TF family.^116^ There are three genes that encode different HIF‐α isoforms: HIF1A, which encodes HIF‐1α; EPAS1, which encodes HIF‐2α; and HIF3A, which is alternatively spliced to generate various HIF‐3α variants in humans.^117^, ^118^ The N‐terminal TAD and oxygen‐dependent degradation domain (ODD) exist in HIF‐1α, HIF‐2α, and variants 1–3 of HIF‐3α, while HIF‐1α and HIF‐2α have an additional C‐terminal TAD.^119^

HIF interacts with and binds to the von Hippel‐Lindau (VHL) protein, activating the ubiquitin ligase system and causing the degradation of HIF by the proteasome under normoxic conditions.^120^, ^121^ Hydroxylation of proline residues in HIFs is required for VHL binding and is mediated by HIF prolyl hydroxylases (also known as prolyl hydroxylase domain, PHD), ketoglutarate‐dependent dioxygenases, and asparaginyl hydroxylase.^122^, ^123^, ^124^ The oxygen sensor proteins PHD and factor inhibiting HIF‐1 become inactive during hypoxia, which causes stabilization of HIF‐α and subsequent dimerization with HIF‐1β and coactivator P300.^125^ HIF dimerizes in the nucleus and binds to E‐box‐like hypoxia response elements, leading to the activation of genes involved in cellular oxygen homeostasis.^126^ Hypoxic cells respond to stress via transcriptional and post‐transcriptional processes, which are primarily controlled by HIF.^127^, ^128^ These molecular modifications enable cells to respond to stress by decreasing oxygen consumption. It has been reported that hypoxia is associated with the production of H_2_O_2_ as a result of mitochondrial ETC inhibition.^129^, ^130^ Chronic intermittent hypoxia, a potentially fatal condition that occurs in several breathing disorders, has also been shown to activate redox signaling, resulting in a variety of systemic and cellular responses.^131^, ^132^, ^133^

### 3.5 ROS generation: The NF‐κB pathway

The NF‐κB family comprises five TFs, including p65/RELA, RELB, c‐REL, p50 (its progenitor p105), and p52 (its precursor p100),^134^ which play essential roles in inflammation,^135^ immunology,^136^ cell proliferation,^137^ and differentiation.^138^ The mature proteins p65/RELA, RELB, and c‐REL possess a C‐terminal TAD within their respective C‐terminal portions.^139^ The TAD area endows p65/RELA, RELB, and c‐REL with the potential to boost the initiation of gene transcription.^140^, ^141^ p105 and p100 lack TAD but possess a CTR containing ankyrin repeats that are cleaved post‐translationally to generate p50 and p52, respectively.^142^, ^143^

NF‐κB activity is tightly controlled due to its potential to regulate the expression of numerous genes. The NF‐κB pathway is primarily regulated by inhibitors of NF‐κB (IκB) and IκB kinase (known as IKK, a kinase that phosphorylates IκB).^144^, ^145^ NF‐κB is maintained inactive in the cytosol during physiological resting states by interacting with an inhibitor of IκB.^146^ IKK phosphorylates IκB, resulting in the ubiquitination and degradation of IκB in response to numerous stimuli, including OS, after which NF‐κB is released and translocates into the nucleus to activate downstream target genes.^147^, ^148^

Since NF‐κB was discovered as a regulator of B‐cell development in 1986,^149^ this family of TFs has been extensively investigated, and aberrant activation of NF‐κB has been found in a wide variety of human disease states.^136^, ^150^, ^151^ In eukaryotic cells, NF‐κB was the first TF to be classified as a redox‐sensitive factor. Staal and colleagues demonstrated for the first time in 1990 that TNF (tumor necrosis factor)‐induced NF‐κB activation is reliant on the intracellular thiol redox state.^152^ One year later in 1991, Schreck and colleagues demonstrated a favorable association between intracellular levels of H_2_O_2_ and NF‐κB activation.^153^ Nonetheless, contradictory evidence about the association between H_2_O_2_ and NF‐κB activation has also been published. Currently, depending on the circumstances, H_2_O_2_ is regarded as either a stimulatory or inhibitory factor for NF‐κB activity. Cytosolic H_2_O_2_ may stimulate the NF‐κB pathway through oxidation and activation of IKK, which functions as a negative regulator of IκB stability.^60^, ^154^ Due to the oxidizable cysteines in NF‐κB, H_2_O_2_ may also directly influence NF‐κB.^155^ Enhanced nuclear H_2_O_2_ accumulation hinders DNA binding, decreasing the transcriptional activity of NF‐κB.^156^ Moreover, increased levels of nuclear Prx1 and Trx have also been reported to promote NF‐κB transcriptional activity.^157^

Although H_2_O_2_ best fulfills the requirements of being a main messenger in redox regulation, other ROS have also been elucidated to function as physiological mediators. For example, O_2_^−•^ activates the ras/rac‐Raf1‐ MAPK and ERK signaling pathways, thus, serving as a growth signal in different cells.^158^ Organic hydroperoxides (ROOH) were reported to act as an essential regulator in cell signaling and determining cell fates.^159^, ^160^ In addition, similar to H_2_O_2_,^1^O_2_ regulates cell fate in a location and dose‐dependent manner. A lower or physiological level of^1^O_2_ can mediate multiple signaling pathways and promote programmed cell death.^161^, ^162^ However, limited by the detection techniques, the underlying mechanisms of these ROS mediating cell signaling are still ambiguous. Further exploration on how diverse ROS regulate intracellular physiological signaling will extend our knowledge of redox biology and help in developing precise redox medicine.

## 4 REDOX IMBALANCE AND OXIDATIVE DAMAGE IN HUMAN DISEASE

Considering the significance of redox signaling in physiological conditions, redox imbalance occurring in different tissues, cells, or organelles may be intimately linked to multiple pathophysiological events. In this section, we focus on several common redox‐relevant diseases, highlighting how precision medicine is now possible by modulating aberrant ROS accumulation and leaving physiological signaling intact.

### 4.1 Type 2 diabetes

The increasing prevalence of diabetes and some related end‐stage organ damage has become a major cause of death and disability around the world.^163^, ^164^ Unfortunately, the underlying mechanisms are still not fully understood, which is a major obstacle in developing targetable antidiabetic therapies. Thus, a deeper understanding of the detailed mechanisms of the initiation and progression of diabetes is an unmet clinical need of great importance for public health.

**FIGURE 3:**
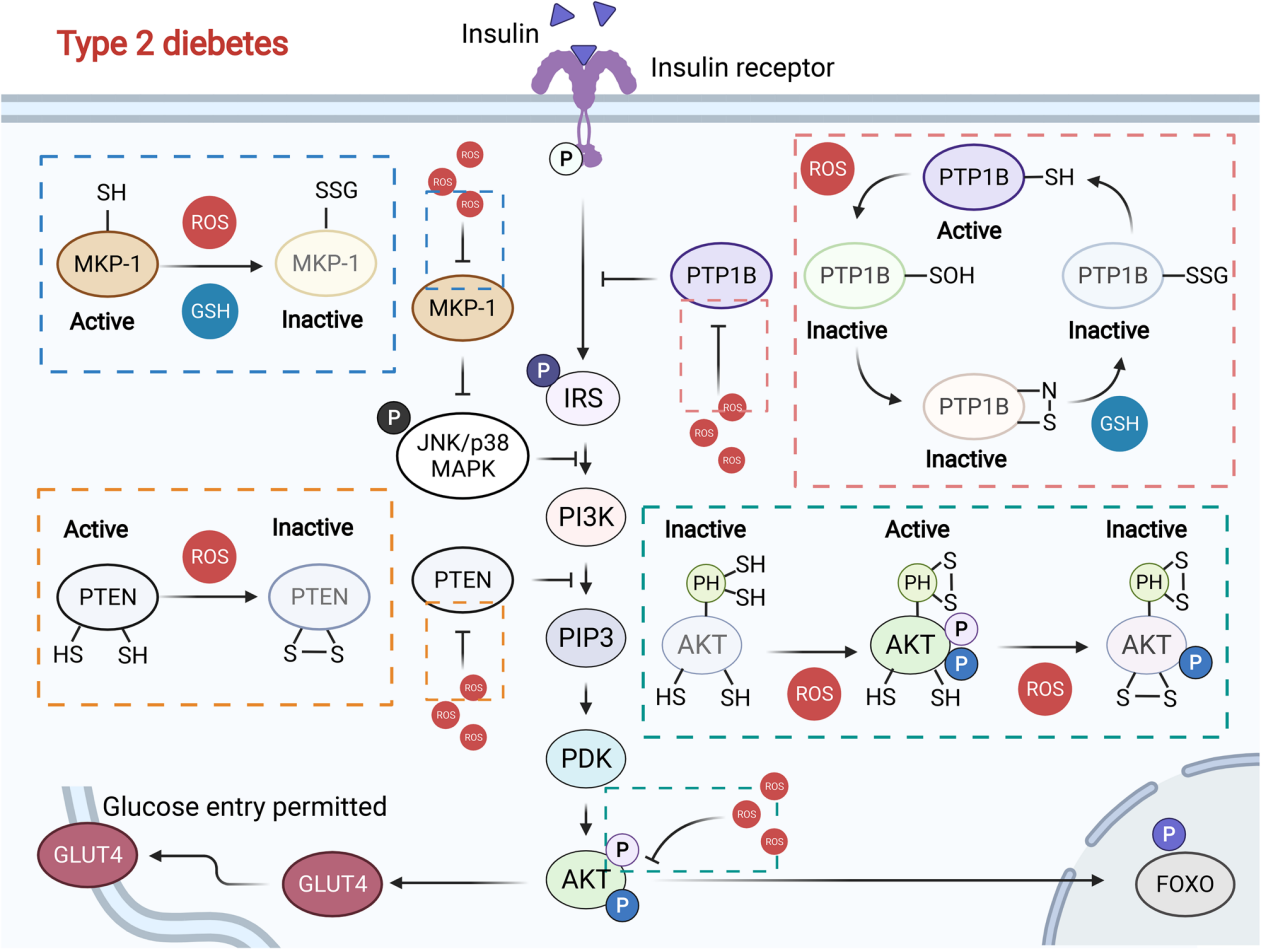
Redox regulation of the insulin signaling pathway. Normally, moderate ROS levels inactivate PTPB1 and PTEN and activate AKT, prompting glucose uptake through GLUT4. However, excessive ROS accumulation leads to glutathionylation modification and proteasomal degradation of MKP‐1, which prevents insulin signaling. Simultaneously, ROS burden also inactivates AKT, inhibiting GLUT4 and subsequent glucose uptake, which eventually causes type 2 diabetes

Mounting evidence has indicated that redox imbalance plays an essential role in diabetes, in which ROS are found to widely influence insulin signaling (Figure 3). Superficially, markers of OS have been largely found at elevated levels in the body fluids of patients with type 2 diabetes, including plasma 8‐hydroxydeoxyguanosine (8‐OHdG, a marker of DNA oxidative damage),^165^ oxidized low‐density lipoprotein (oxLDL) to LDL ratio,^166^ GSH conjugation to haemoglobin,^167^ 8‐iso‐PGF2‐α,^168^ protein carbonyls,^169^ urine 8‐OHdG, and 8‐iso‐PGF2‐α.^170^ However, to date small‐molecule antioxidant drugs have exhibited poor outcomes, suggesting that a different redox‐based perspective should be urgently sought. In recent years, important conceptual breakthroughs have attracted much interest, turning “general redox biology” into “precise redox biology” on the basis of increased knowledge of the disease‐relevant ROS sources and physiological roles of ROS. The increased ROS levels mainly arise from dysfunctional mitochondria^171^ and NOX1,^172^ which are accompanied by the common symptoms of diabetes—hyperglycemia and dyslipidemia. Therefore, more targetable therapeutic strategies are now available for exploitation.

Multiple key regulators and targets of insulin receptor (IR) signaling have been revealed to be redox‐sensitive. Their aberrant forms are extensively found in type 2 diabetes,^173^, ^174^ including AKT, protein tyrosine phosphatase 1B (PTP1B), phosphatase and tensin homologue (PTEN), JUN amino‐terminal kinase (JNK), and FOXO. Insulin binds to the IR, phosphorylating IR substrate proteins (e.g., IRS1 and IRS2) and activating the phosphatidylinositol 3‐kinase (PI3K)‐AKT signaling pathway, which eventually promotes the translocation and activation of glucose transporter 4 (GLUT4) and glucose uptake.^175^, ^176^ PTP1B, a negative regulator of IR, dephosphorylates important tyrosine residues of IR and is tightly regulated by its in vivo redox status.^175^ Physiologically, a low level of ROS was found to increase insulin sensitivity by modulating stress‐response kinases and further dephosphorylation and decreased activity of PTP1B and PTEN, which dephosphorylated IR and downregulated the PI3K signaling pathway, respectively.^175^, ^176^ Simultaneously, NOX4‐mediated ROS activated MAP kinase phosphatase‐1 (MKP‐1), which indirectly led to reduced IRS‐1 phosphorylation on serine residues, thereby increasing IRS‐1 tyrosine phosphorylation by attenuating ERK1/2 signaling.^177^ Nevertheless, high levels of ROS potently promote insulin resistance and are associated with several abnormalities in type 2 diabetes including hyperglycemia, increased nonenzymatic glycosylation, inflammation, and activation of ETC production.^178^

As a major insulin resistance mechanism, high ROS levels prompted the activation of JNK, which largely outweighed PTP1B inactivation.^179^ ROS activated JNK and p38 by modulating their regulatory proteins. A representative example is that MKP‐1 inactivated JNK and p38 MAPK by dephosphorylation. However, the process can be manipulated by glutathionylation modification, which targeted MKP‐1 for proteasomal degradation.^180^ The FOXO family of TFs has four main members—FOXO1, FOXO3, and FOXO4, which share similar properties, and FOXO6, which occupies a different expression form. FOXOs have several cysteine residues that are highly sensitive to redox stress and are conducive to producing antioxidants and relieving ROS overload.^181^ However, hyperactivation of FOXOs significantly induces hypertriglyceridemia, hyperglycemia, and insulin resistance that eventually cause incurable diabetes.^182^, ^183^ There is a compensatory process of the insulin signaling pathway to target FOXOs. Phosphorylating and blocking them in the cytoplasm with ROS‐modulated PI3K‐AKT decreased DNA binding to its consensus response elements and increased nuclear exclusion.^174^

### 4.2 Atherosclerosis

In pathological conditions of atherosclerosis, plaque appears in the intimal layer of arteries, which persistently accumulates and eventually causes stroke and infarction.^184^ Redox disease—diabetes is an important inducer of the initiation of atherosclerosis, and accumulating evidence also reveals that oxidant burden plays an important role in atherosclerosis.^185^

Similar to type 2 diabetes, multiple markers of OS were shown to have elevated levels in patients suffering from atherosclerosis. For instance, lipid hydroperoxides were first identified in human atherosclerotic aortae in 1952.^186^ Following that, increasing research has shown that oxidized lipids and other oxidant markers aggregate in atherosclerotic lesions. In freshly isolated human atherosclerotic plaques, 20% of cholesteryl linoleate was oxidized, in contrast to undetectable levels in normal arteries.^187^ OS was reported to function in the conversion of LDL into oxLDL, and modified LDL was considered a pivotal marker of the initiation and development of atherosclerosis.^188^ For example, 4‐hydroxy‐2‐nonenal (HNE)‐modified LDL was elevated by 50% in the plasma of patients with atherosclerosis compared with healthy volunteers.^189^ Malondialdehyde (MDA), the product of lipid peroxide that originates from prostanoid metabolism, is the other modification pattern of LDL.^188^ Circulating MDA‐LDL levels have also been identified as a marker of ROS overload in atherosclerosis and are elucidated to be closely related to the prognosis of coronary artery disease.^190^, ^191^, ^192^ In addition, isoprostanes, as peroxidation products of arachidonic acid, were proven to be fivefold higher in atherosclerotic lesions than in umbilical veins.^193^ All of these oxidant markers confirm that atherosclerosis may also be a redox‐relevant disease.

**FIGURE 4:**
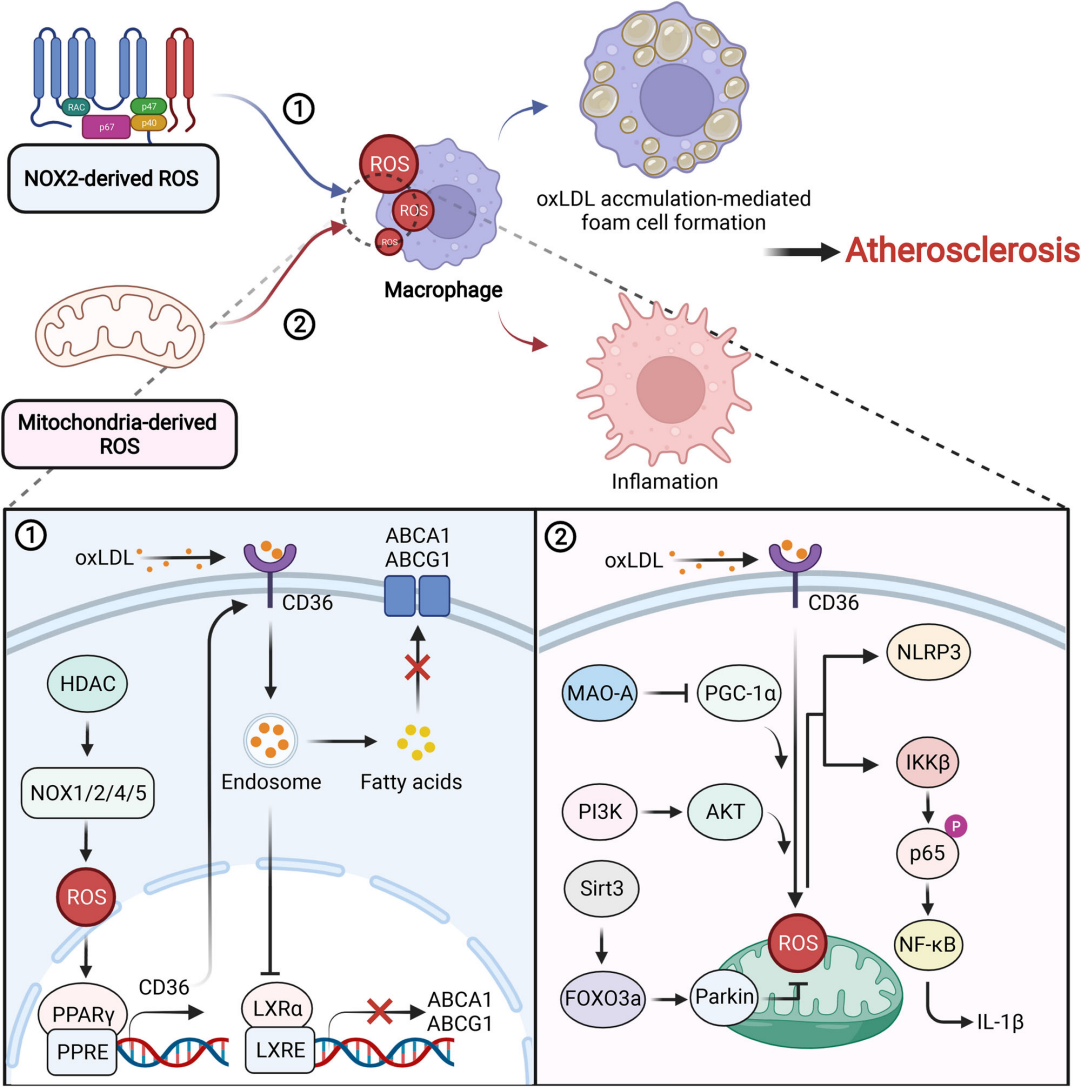
The roles of NOX and mitochondria‐derived ROS in atherosclerosis. NOX‐derived ROS promote CD36 expression, which facilitates the transportation of oxLDL and inhibition of ABCA1 and ABCG1 transcription. Under these conditions, fatty acids accumulate in the cytoplasm that engenders macrophage transforming into foam cells. On the other hand, mitochondria‐derived ROS elicit the production of NLRP3 and IL‐1β through multiple signaling pathways, accounting for an inflammatory phenotype in atherosclerosis pathogenesis. Both of them are important inducers of atherosclerosis

How ROS contribute to the initiation and development of atherosclerosis is a vital question worthy of exploration. Multiple underlying mechanisms have been proposed on the basis of its sources—NOXs and mitochondria (Figure 4).^194^, ^195^ Among NOXs, NOX4 is abundantly found in the plagues of atherosclerosis, which was reported to correlate with atherosclerosis in 2002.^196^ NOX4 is a double‐edged sword in atherosclerosis that can both relieve and exacerbate the disease. In mouse models of atherosclerosis, NOX4‐derived ROS were essential for vessel homeostasis. Two NOX4‐knockout models—NOX4^–/–^/Ldlr^–/–^ and NOX4^–/–^/ApoE^–/–^ mice, suffer remarkable endothelial damage and plaque burden.^197^, ^198^ In contrast, global NOX4 deletion can both protect against and aggravate diabetes‐induced atherosclerosis, depending on the time frame.^199^ Evidence has shown that NOX4 deletion promoted diabetes‐induced plaque formation in the early stage, but in the progressive stage, NOX4 deletion helped to repress inflammation.^200^ Besides, NOX4 can increase plaque burden with less T‐cell activation and infiltration in a 10‐week model of diabetes, but conversely, in the 20‐week model of diabetes, NOX4 can decrease collagen deposition and proliferation, blocking advanced lesions.^199^, ^201^ In addition to NOX4, other NOXs have also been found to be aberrantly upregulated in atherosclerosis. For example, NOX1 was considered an important risk factor in OS‐mediated inflammation and atherosclerosis.^172^ NOX2 was found to have a particular function, which enabled the recruitment of macrophages through ROS‐vascular cell adhesion molecule‐1 signaling and the activation of endothelial cells.^202^ Chen et al. revealed that NOX2 could also upregulate peroxisome proliferator‐activated receptor activity and CD36 expression by augmenting ROS levels, accelerating the formation of macrophage‐foam cells, and development of atherosclerosis.^203^ Calcium‐dependent NOX5 was also a source of ROS, reflecting a marked increase and possible application potential in atherosclerosis.^204^ Intriguingly, global inhibition of NOXs transcription via histone deacetylase (HDAC) blockade dramatically relieved OS and inflammation, suggesting that HDAC was upstream of NOXs and could become a good target for atherosclerosis.^205^ Moreover, NOXs increased ROS levels, helping internalize oxLDL into macrophages through CD36 and generating foam cells, which may be a universal mechanism underlying NOX‐mediated progression of atherosclerosis judging from available reports.^203^, ^206^

Mitochondrial ROS (mitoROS)‐mediated aberrant signaling has been largely elucidated to accelerate atherosclerosis and causes associated vascular complications, which are frequently accompanied by plaque accumulation.^207^, ^208^, ^209^ Elimination or genetic inhibition of mitoROS prevented the development of atherosclerosis and associated complications. Melatonin was capable of efficiently scavenging mitoROS through the Sirtuin3 (Sirt3)/FOXO3a/Parkin signaling pathway, preventing the production of the nucleotide‐binding domain and leucine‐rich repeat pyrin domain containing 3 (NLRP3), thus, largely ameliorating atherosclerosis.^210^ Besides, renal denervation, an agent that inactivates mitochondrial monoamine oxidase A and subsequently peroxisome‐proliferator‐activated receptor‐γ coactivator‐1α (PGC‐1α), was able to attenuate mitoROS‐mediated inflammation and atherosclerosis.^211^ In wild‐type (WT) macrophages, LPS and oxLDL‐induced mitoROS remarkably strengthened IKK‐β and downstream p65 phosphorylation which could be inhibited by overexpressing mitoROS‐targeted catalase that efficiently repressed attenuated lesion progression and immune cell infiltration.^212^ Another publication described PI3K/AKT was upstream of mitoROS, and blocking it may be an efficient method for treating atherosclerosis.^213^ The ROS scavenging system comprises many regulatory proteins, among which the proteins of paraoxanase (PON) family were able to resist mitoROS in the development of atherosclerosis due to their capacity to hydrolyze lipid peroxides.^214^, ^215^, ^216^ Overexpression of the PON1/2/3 cluster facilitated collagen synthesis, narrowed the necrotic core area, and decreased oxLDL and inflammatory markers, inhibiting mitochondrial dysfunction and stimulating plaque stability to alleviate atherosclerosis.^217^, ^218^ PON1 can also inhibit monocyte‐to‐macrophage differentiation and help macrophages resist oxLDL‐induced foam cell formation,^219^ which prevented the formation of an inflammatory phenotype.^220^ In contrast, PON1 deficiency was frequently observed in lesions of atherosclerosis, which resulted in vascular OS and leukocyte adhesion. Therefore, downregulation of PON expression may be a prerequisite for atherosclerosis.^221^

### 4.3 Chronic obstructive pulmonary disease

Incurable chronic obstructive pulmonary disease (COPD) is characterized by progressive dyspnea and functional loss of the lung, and is a major public health issue.^222^ COPD includes progressive chronic bronchitis and emphysema, often eliciting associated complications, including lung cancer, cardiovascular disease, skeletal muscle wasting, and osteoporosis.^223^, ^224^, ^225^, ^226^ Induction of COPD has many causes, including OS, inflammation, protease antiprotease imbalance, and apoptosis,^227^, ^228^, ^229^ among which OS is probably the fundamental mechanism due to its central roles in other processes. Likewise, several oxidized products or markers of OS have been discovered in patients with COPD. Increasing ROS generation was found in the airways of patients with COPD, with superoxide and MDA being detected in the blood, sputum, airspaces, and lungs.^230^, ^231^ 8‐Isoprostane was also consistently detectable in the exhaled breath condensate of COPD patients compared with healthy controls.^232^ In addition, HNE was abundantly found in the airways and alveolar epithelial cells, endothelial cells, and neutrophils, with levels being elevated by at least 50% in patients with COPD.^233^ Simultaneously, in urinary samples from COPD patients, the level of 8‐OHdG was also dramatically increased.^234^ Furthermore, ROS levels were significantly elevated with COPD exacerbation, and the level of OS was inversely associated with the lung function of patients with COPD, hinting that oxidant burden may be an essential risk factor in the initiation and progression of the disease.^233^, ^235^

**FIGURE 5:**
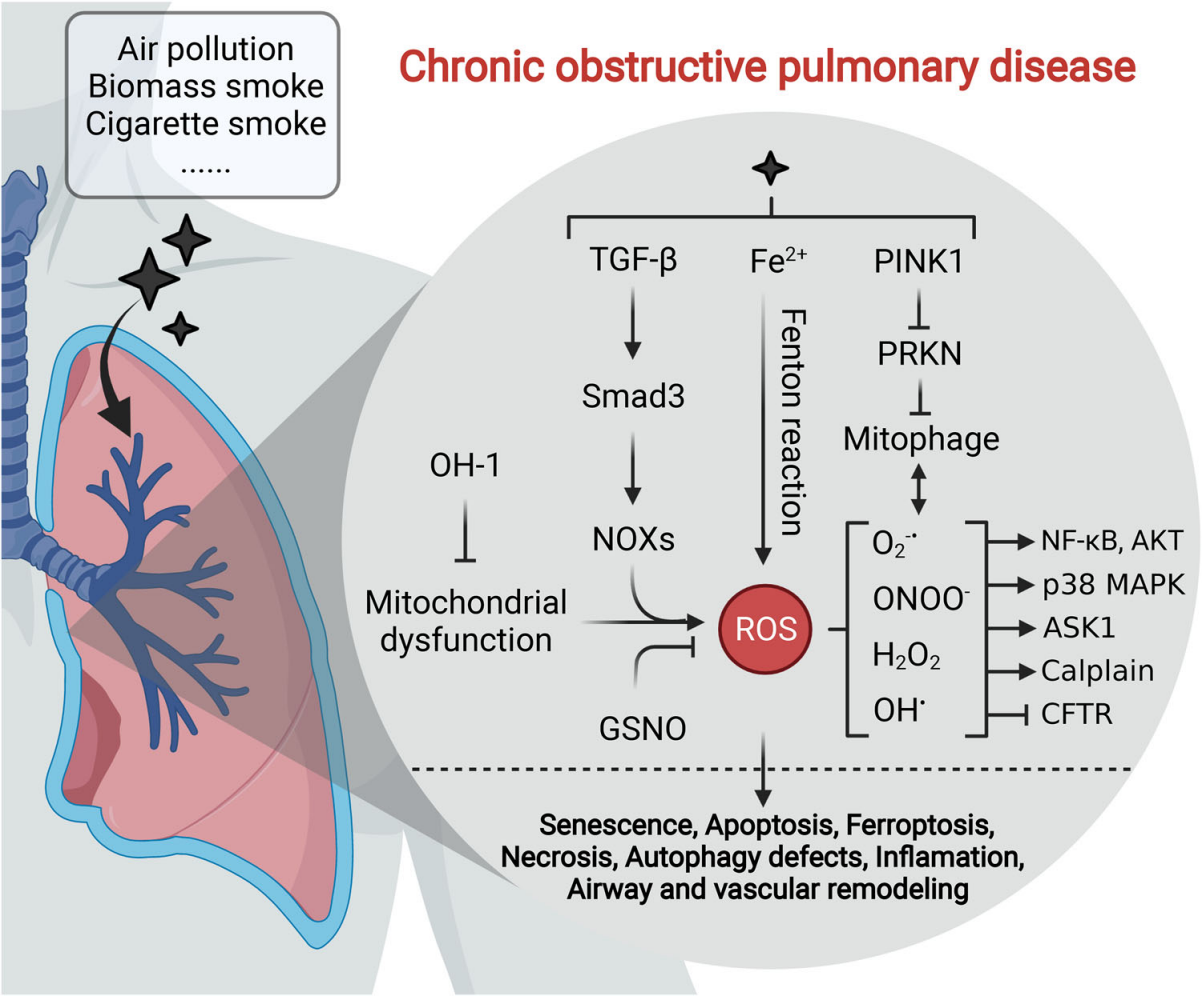
Exogenous and endogenous factor‐induced ROS mediate COPD pathogenesis. Exogenous sources (i.e., air pollution, biomass smoke, and CS) and endogenous sources (i.e., mitochondria and NOX) are important inducers of COPD, which accelerate various ROS accumulation in the lung through multiple pathways including TGF‐β signaling, Fenton reaction and mitochondrial dysfunction, stimulating cell senescence and death, autophagy defects, inflammation and airway, and vascular remodeling

Sources of oxidants in patients with COPD originate from both endogenous and exogenous elements (Figure 5). For exogenous sources, cigarette smoke (CS), biomass smoke, and air pollution are the main causes of COPD, which can generate various ROS, including O_2_^−•^, ONOO^−^, H_2_O_2_, and OH•.^222^, ^236^ Airway and pulmonary vascular remodeling are two characteristics of COPD. Zhu et al. disclosed that CS‐induced ROS‐activated calpain, which led to COPD exacerbation through the airway and pulmonary vascular remodeling.^237^ OS‐mediated mitochondrial dysfunction was also a cause of airway remodeling and inflammation in COPD.^233^ Additionally, ROS stimulated the overgrowth of airway smooth muscle through ASK1/JNK/P38 MAPK pathway.^238^ ROS impaired the expression and function of CTFR, which was another contributor to CS in COPD.^239^ Notably, CTFR dysfunction could be reversed by S‐nitrosoglutathione, which restored autophagy impairment and alleviated chronic inflammatory‐OS in CS‐induced COPD.^240^ Consistently, insufficient mitophagy resulted in PTEN‐induced putative protein kinase 1 (PINK1) accumulation and parkin RBR E3 ubiquitin protein ligase reduction via PINK1‐mediated proteasomal degradation, which further accelerated insufficient mitophagy and ROS release, becoming a significant inducer of COPD pathogenesis.^241^ Additionally, a similar study revealed that CS impaired PARK2 function and mitophagy, causing ROS‐mediated cell senescence. Conversely, severe CS exposure induced excessive mitophagy, contributing to apoptosis and necrosis of primary human bronchial epithelial cells.^242^ Intriguingly, CS induced the accumulation of iron in mitochondria and cytosol, following which mitoROS and lipid peroxidation occurred that further engendered necrosis and ferroptosis.^243^ Exposure to cigarette smoking evoked inflammatory responses and related chain reactions, such as cell death and fibrosis, which are important to the development of COPD.^244^ Additionally, CS‐induced OS significantly reduced phagocytosis of macrophages from COPD, which was linked to enhanced inflammation.^245^

Endogenous sources—mitochondria, NOXs, myeloperoxidase (MPO), and inducible nitric oxide synthase (iNOS)—also play pivotal roles in COPD.^244^ The levels of mitoROS were elevated in patients and animal models with COPD, which was proven to be an inducer of inflammation and airway remodeling.^246^ Heme oxygenase protected mitochondria from mitochondrial dysfunction, decreasing OS‐mediated senescence of lung fibroblasts that was closely linked with COPD.^247^ In COPD, a study was conducted to explore the specific sites of mitoROS generation, in which complex III was identified as a main site of mitoROS production, and seemed to closely correlate with muscle oxidative damage.^248^ NOXs are major intracellular sources of abundant ROS and are composed of multiple subtypes, as discussed earlier. Accumulation of NOX‐derived ROS has been largely found in diverse chronic diseases of the respiratory system, which ultimately progress to COPD.^249^, ^250^ For example, the NOX‐ROS‐NF‐κB transduction pathway was observed to be involved in the pathogenesis of patients with COPD.^251^ NOX4 was a contributor to multiple lung diseases, including acute respiratory distress syndrome, pulmonary fibrosis, and pulmonary vascular disease, leading to COPD and cancer.^252^ Airway and alveolar epithelial cells aberrantly express NOX1/4, resulting in the progression of acute lung injury, neutrophilic asthma, and pulmonary fibrosis.^253^, ^254^, ^255^ Besides, Smad3‐NOX4‐derived ROS‐mediated p38 MAPK/AKT signaling was involved in TGF‐β (transforming growth factor)‐induced airway remodeling, which may be a general mechanism underlying COPD pathogenesis.^256^

### 4.4 Alzheimer's disease

Among the many widely studied neurological disorders, neurodegenerative diseases, such as Alzheimer's disease (AD),^257^ Parkinson's disease (PD),^258^ Huntington's disease (HD),^259^ and multiple sclerosis^260^ are closely related to redox imbalance. Intracellular mitochondrial dysfunction and excitotoxicity of the brain and spinal cord frequently occur in these diseases, directly resulting in apoptosis and functional loss of nerve cells and increased risk of these diseases.

AD is characterized by the accumulation of extracellular amyloid β‐peptide (Aβ) plaques and intracellular neurofibrillary tangles (NFTs).^257^, ^261^ Multiple risk factors, such as genetics,^262^, ^263^ environment,^264^, ^265^ diet,^266^, ^267^ age,^268^, ^269^ sex,^270^, ^271^ and race,^272^ have been confirmed in AD, but the underlying mechanisms remain largely unknown. Many studies have illustrated that increased OS occurs in different parts of brains in patients with AD, including elevated levels of F2‐isoprostane‐α in cerebrospinal fluid and frontal and temporal poles,^273^, ^274^ acrolein in the amygdala and hippocampus/parahippocampal gyrus,^275^ and HNE in ventricular fluid, hippocampus, inferior parietal lobule, and cortex.^276^, ^277^, ^278^ Additionally, nuclear and mitochondrial DNA oxidation were also observed in the frontal, parietal, and temporal lobes of the brain in AD patients.^279^ Hence, we can justifiably conclude that OS may play a crucial role in AD through the involved signaling pathways.^257^

Oxidant burden is derived from multiple causes in the brains of patients with AD, such as Aβ, activated microglia, iron accumulation, and dysfunctional mitochondria.^280^, ^281^, ^282^, ^283^ To relieve ROS overload, Nrf2 was considered as a “dark horse” in AD treatments, as a decline in Nrf2 function was frequently observed in patients with AD, and Nrf2 activation exhibited potent therapeutic potential in several AD models.^284^ Nrf2 deficiency in an AD mouse model exacerbates spatial learning and memory disorders.^285^ Notably, Nrf2 knockout mice also displayed a similar mRNA pattern as patients with AD.^286^ Eukaryotic elongation factor‐2 kinase was upregulated in patients with AD, which was found to negatively regulate Nrf2 and lead to Aβ generation.^287^ The interaction between inhibitor of apoptosis‐stimulating protein of p53 (iASPP) and Keap1 can stabilize Nrf2 and restore cellular redox balance.^288^ Mounting evidence has revealed that glycogen synthase kinase (GSK)‐3β activity is involved in phosphorylation‐mediated Nrf2 degradation.^289^ Increased expression of GSK‐3β has been observed in different studies on AD.^290^, ^291^ GSK‐3β phosphorylation and inhibition via AMPK or PI3K/AKT favored an increase in Nrf2 expression, which significantly relieved Aβ‐induced oxidative damage.^292^, ^293^, ^294^ β‐secretase enzyme (BACE1) is responsible for the generation of Aβ, and a slight increase in BACE1 expression contributes to remarkable Aβ accumulation. In patients with AD, BACE1 is aberrantly upregulated and positively correlates with the levels of Aβ. A noteworthy example showed that Nrf2 exerted an essential role in the BACE1‐Aβ axis.^284^ Nrf2 bound to the ARE in the promoter of BACE1, suppressing its expression in an animal model of AD and reducing subsequent Aβ generation.^295^ As a result, Nrf2 activation ameliorated cognitive deficits. By contrast, Nrf2 deletion significantly elevated BACE1 and Aβ levels, exacerbating cognitive deficits. Nrf2 stabilization increased SOD1 synthesis and inhibited the NF‐κB‐NOX2‐ROS axis, which prevented BACE1 expression and Aβ production.^296^

**FIGURE 6:**
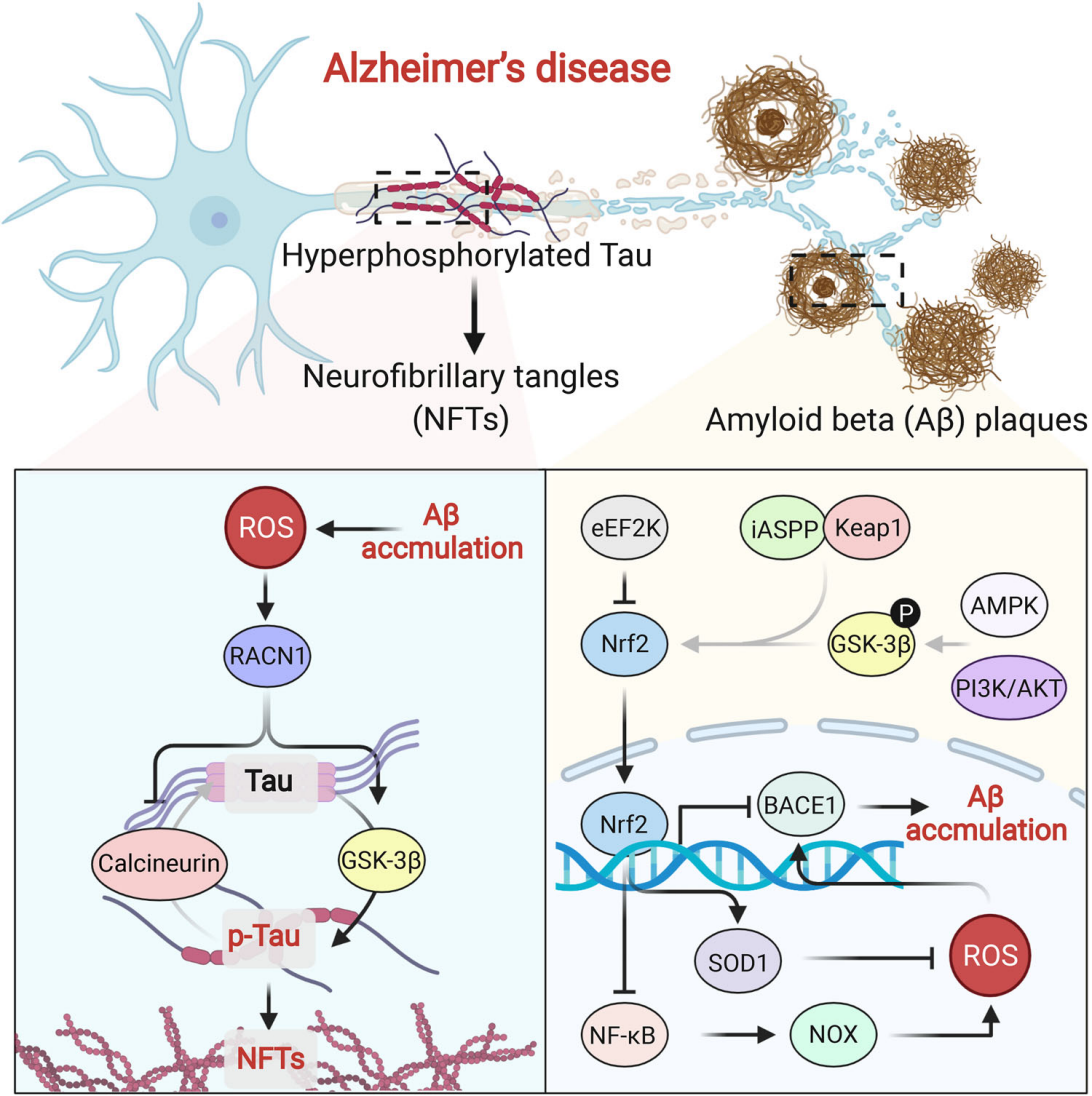
ROS accelerates Aβ accumulation and p‐tau formation in AD. Phosphorylated tau protein (p‐tau) and Aβ are essential risk factors in AD, which originate from the downregulated Nrf2 and increased ROS burden. Dysregulated Nrf2 is controlled by different proteins, promoting ROS overproduction and BACE1 expression which elicits Aβ accumulation. Aβ accumulation further causes ROS generation and activates RACN1, phosphorylating tau protein through calcineurin and GSK‐3β

Misfolding of the tau protein causes tauopathies and accelerates the production of β‐sheet fibrils and NFTs, which has been proven to be an important risk factor for AD.^297^, ^298^, ^299^ Overexpression of GSK‐3β caused neuronal loss and memory disorders, which may be the result of tau hyperphosphorylation.^300^, ^301^ PINK1 was capable of restoring the activity of PI3K/AKT/GSK3β signaling, stabilizing Nrf2, and alleviating tau hyperphosphorylation.^302^ Similarly, direct inhibition using antisense oligonucleotide of GSK‐3β also activated Nrf2, reducing the phosphorylated tau protein and leading to improved learning and memory in animal models.^303^ Intriguingly, an inhibitor—dimethyl fumarate, can simultaneously prevent both GSK‐3β and Keap1 activity in a preclinical model.^304^ As expected, dual inhibition potently activated Nrf2, which provided a promising method for reducing tau phosphorylation and treating neurodegenerative diseases including AD. Interestingly, Aβ‐induced ROS were an important cause of tau activation and AD exacerbation, which was mediated by the regulator of calcineurin gene (RCAN1) synthesis and subsequent calcineurin inactivation and GSK‐3β activation.^305^ A schematic outlining Aβ accumulation and tau phosphorylation has been presented in this text (Figure 6).

Autophagy has been extensively reported to be involved in the clearance of tau protein. p62 is a pleiotropic protein involved in multiple intracellular activities, in which the autophagic degradation of aggregated proteins is mostly reported.^284^ p62‐knockout mice exhibited neurodegenerative features, indicating that p62 may be involved in the initiation and progression of AD. Using an animal model of AD, Zheng and colleagues revealed that p62‐Keap1‐Nrf2 signaling was involved in this process, with p62 degradation downregulating Nrf2 expression.^306^ These data suggest that p62 may be a pivotal upstream protein that regulates tau hyperphosphorylation and largely influences the development of AD. Gu et al. also reported that activation of p62 could overcome Aβ‐induced cell death, which was mediated by the activation of Nrf2 and autophagy.^307^ In addition, Xu and colleagues demonstrated that p62 controlled autophagic clearance of pathogenic microtubule‐associated proteins tau, which dramatically reduced neurofibrillary tangle accumulation and pathological spreading.^308^ Nuclear dot protein 52 (NDP52), an autophagy adaptor protein, is capable of efficiently promoting autophagic degradation of p‐tau. Jo et al. found that Nrf2 decreased p‐tau levels and alleviated AD by inducing NDP52 expression.^309^ Mechanistically, Nrf2 bound to AREs in the promoter of NDP52 and enhanced its transcription. Another study also suggested a similar function of Nrf2 in clearing p‐tau and relieving AD. Nrf2, as a key regulator, modulated selective autophagy that helped to eliminate tau species, which was mediated by the expression of p62, NDP52, NBR1, and BAG3.^310^ Additionally, Kim and colleagues illustrated the detailed mechanisms of Nrf2, TFEB, p62, and NDP52 in autophagic elimination of tau protein.^311^ In support of the large application potential of Nrf2 in AD, various Nrf2 activators like benfotiamine and sulforaphane have shown excellent therapeutic effects on the basis of inhibiting the tau protein.^312^, ^313^

### 4.5 Cancer

The hallmarks of cancer involve redox alterations, which are complex based on distinct stages in cancer progression.^314^, ^315^, ^316^ ROS are involved in each stage of tumorigenesis including the malignant transformation of normal cells, metastasis, and resistance to therapy.^317^, ^318^, ^319^, ^320^ The underlying mechanisms mainly result from the disruption of physiological redox signaling, producing pro‐oncogenic and antiapoptotic signals in cancer initiation and progression. Increasing ROS levels in cancer cells greatly strengthen proliferation and metastasis by regulating multiple signaling pathways and TFs,^321^ and under these conditions, metabolic reprogramming also occurs in response to nutrition deficiency and hypoxia.^322^, ^323^, ^324^ Similar to other redox‐relevant diseases, various oxidants have been widely detected in the body fluids of cancer patients including elevated H_2_O_2_ levels in patients with non‐small‐cell lung cancer and increased levels of 8‐OHdG in patients with prostate cancer and lung cancer.^325^, ^326^, ^327^

ROS‐modulated expression of oncogenes and alterations of involved signaling pathways have been extensively reported to be associated with cancer initiation. As a molecule downstream of WNT signaling, RAC1 was found to potently trigger ROS production and activate the ROS‐NF‐κB pathway, leading to the proliferation of intestinal stem cells and colorectal cancer initiation.^328^ Myeloid‐derived H_2_O_2_ induced genome mutations in intestinal epithelial cells, initiating the malignant transformation of normal epithelium. Furthermore, H_2_O_2_ also drove additional mutations in transformed epithelial cells, evoking cancer metastasis through the NF‐κB‐AKT‐TNFα‐H_2_O_2_ feedback loop.^329^ The accumulation of self‐renewing tumor‐initiating cells is a primary driving force for thyroid cancer. An inspiring study found that in CD133^+^ self‐renewing tumor‐initiating cells, NOX1 was phosphorylated and activated by STAT3, and subsequent ROS overload stimulated the PI3K/AKT pathway, which increased the self‐renewal activity and tumorigenicity of CD133^+^ thyroid cells.^330^ Notably, reducing ROS levels may also be a cause of cancer initiation. Cheung et al. reported that ROS could be dynamically regulated by TIGAR, an antioxidant protein, in different phases of tumorigenesis.^318^ In premalignant lesions, a higher level of TIGAR resulted in a lower ROS level, which was conducive to cancer initiation. However, in metastasizing tumors, TIGAR was downregulated and ROS were abundantly generated, decreasing dual‐specificity phosphatase 6 expression to activate ERK signaling and drive metastasis.

**FIGURE 7:**
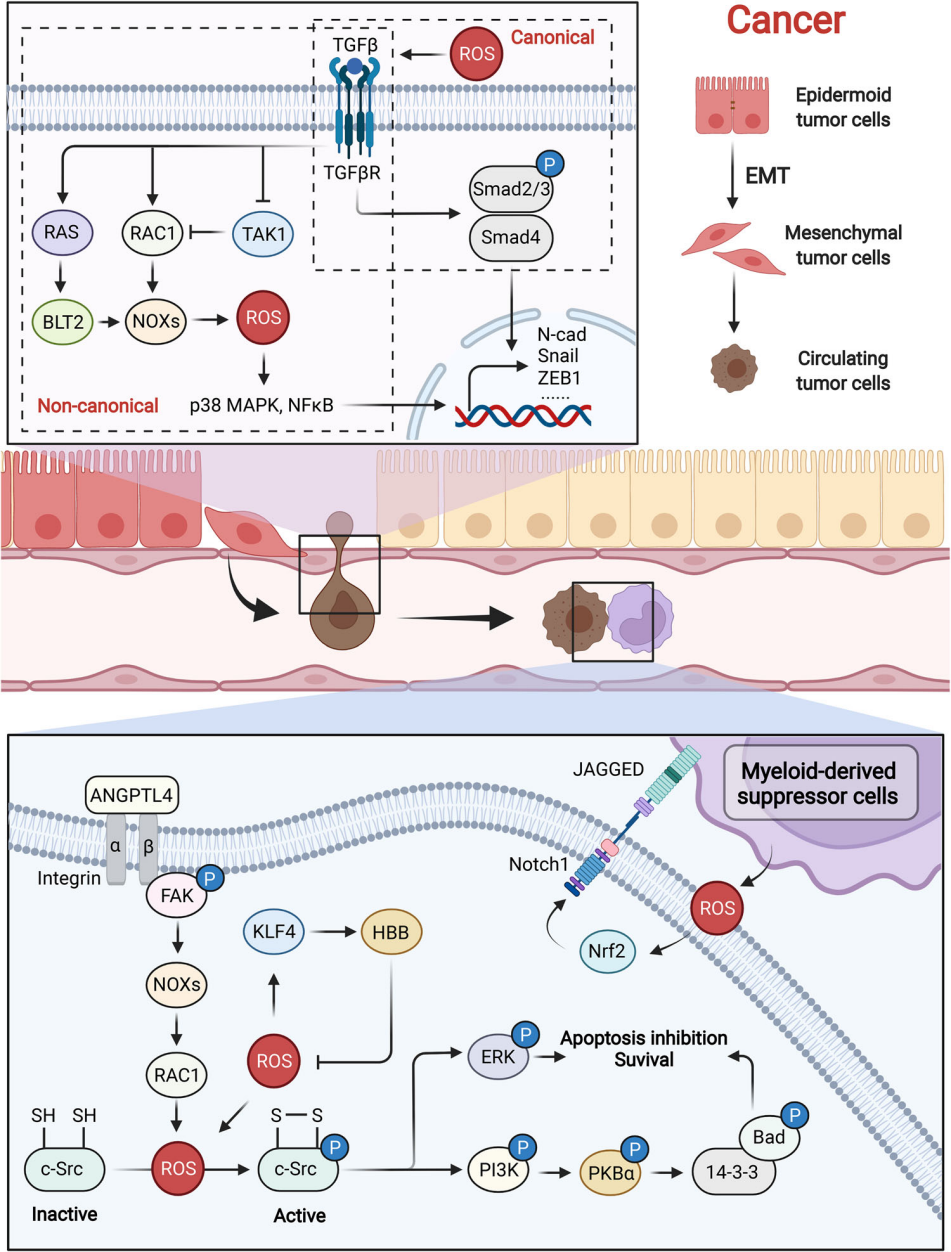
ROS promote the EMT of epithelial cells and survival of CTCs during cancer metastasis. During cancer metastasis, extracellular and intracellular ROS activate TGF‐β signaling and trigger an EMT phenotype through canonical and noncanonical pathways. CTCs entering the blood stream also face ROS stress, which protects them from apoptosis and immune surveillance

Metastasis, the spread of primary cancer cells to distant organs and coupled with drug resistance, has become the main cause of cancer patient death. In solid cancers, metastasis generally begins with epithelial‐mesenchymal transition (EMT),^331^ leading to the detachment and infiltration of primary tumor cells from the basement membrane into the local vasculature and/or lymphatics. Circulating tumor cells (CTCs), which enter the systemic circulation and evade anoikis and immune surveillance, is the other prerequisite for colonization of distal organs. These processes are largely regulated by altered redox status (Figure 7). ROS‐mediated TGF‐β signaling has been extensively reported to be involved in EMT, leading to cancer metastasis.^332^ ROS dynamically interact with TGF‐β, modulating EMT through multiple signaling pathways.^332^ Canonical TGF‐β signaling was mediated by phosphorylated Smad2/3 proteins that entered the nucleus and activated Smad4, which activated the EMT process. In addition, noncanonical pathways mediated by RAC,^333^ RAS, MAPK,^334^ and TGF‐β‐activated kinase 1^335^ signaling also play essential roles in ROS‐mediated EMT. The fate of CTCs followed by the detachment of primary cancer cells from the basement membrane is also determined by ROS. CTCs are capable of resisting anoikis, which largely depends on redox regulation. An elegant study demonstrated that tumor‐derived angiopoietin‐like 4 protein (ANGPTL4) specifically bound to integrins and subsequently activated FAK and Rac1, which further prompted NOX‐dependent ROS generation. Increased ROS oxidized the redox sensor c‐Src, stimulating downstream PI3K and ERK‐mediated survival and preventing Bad‐mediated apoptosis.^336^ However, excessive ROS burden facilitated CTC death without KLF4‐mediated induction of β‐globin (HBB).^337^ In the tumor microenvironment, myeloid‐derived suppressor cells (MDSCs) inhibit the toxic effects of T cells and further accelerate cancer progression. Intriguingly, MDSCs were also found to promote cancer metastasis by regulating CTCs. Mechanistically, MDSCs interacted with CTCs, which aided MDSC‐derived ROS to elevate Notch1 receptor expression in CTCs via the ROS‐Nrf2‐ARE axis. Jagged1‐expressing MDSCs contributed to the activation of Notch signaling in CTCs by engaging the Notch1 receptor, leading to the dissemination and metastasis of CTCs.^338^

Cancer therapeutic resistance is the other main reason for treatment failure and patient death. Accumulating research has found that the metabolic shift from glycolysis toward mtOXPHOS is an important cause of cancer chemoradiotherapy resistance.^339^ In chemotherapy‐resistant breast cancer stem cells (CSCs), MYC and MCL1 were upregulated and cooperated to promote mtOXPHOS, exerting a resistant role to chemotherapy via HIF‐1α.^340^ OXPHOS is essential for maintaining the stemness of cancer cells, which can be targeted to exploit anticancer drugs. The antidiabetic drug metformin, a well‐established agent for mitochondrial inhibition, has been proposed to benefit cancer therapy.^341^ However, drug resistance frequently occurs, which may be due to the MYC/PGC‐1α balance. Moderate levels of MYC/PGC‐1α prompted a plastic phenotype, an intermediate state between differentiated cancer cells and CSCs. MYC prevention through a BET inhibitor augmented PGC‐1α expression, engendering the stemness of cancer cells that can be further killed by metformin.^342^ Venetoclax, an FDA‐approved BCL2 inhibitor, has achieved some success in managing lymphoid malignancies, but resistance to this drug is emerging. A study revealed the underlying mechanisms that involved apoptotic and metabolic pathways simultaneously, implying multiple possible strategies for reversing drug resistance.^343^ Likewise, under venetoclax treatment, monocytic subclones were shown to embrace a specific transcriptome profile, which downregulated BCL2 and triggered MCL1‐mediated OXPHOS and survival.^344^ In addition, venetoclax is also an inhibitor of amino acid metabolism, exerting a killing effect on leukemia stem cells. Treatment failure of venetoclax targeting amino acid metabolism may partially be attributed to nicotinamide metabolism. The nicotinamide phosphoribosyltransferase inhibitor is a potent agent for reversing venetoclax resistance.^345^

### 4.6 Aging/lifespan

**FIGURE 8:**
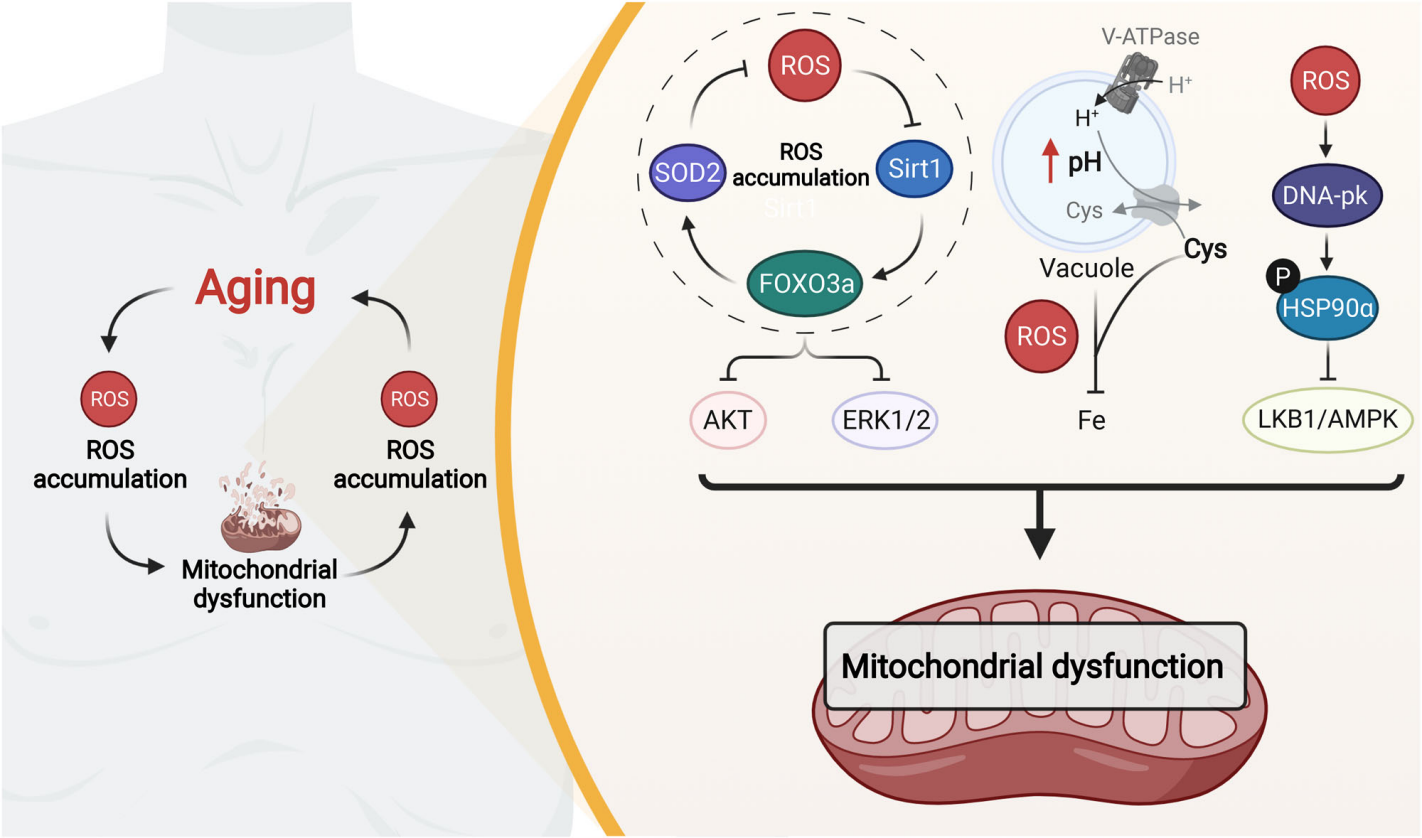
The positive feedback loop of the aging‐ROS‐mitochondrial dysfunction‐ROS axis. Aging generally accompanies ROS overproduction, which contributes to mitochondrial dysfunction via different mechanisms. Notably, mitochondrial dysfunction further induces ROS generation and accelerates aging, suggesting that scavenging ROS could efficiently attenuate aging

As reviewed above, dysregulated redox homeostasis is intimately associated with aging‐related diseases such as AD and cancer. Aging itself involves altered redox status, whose hallmarks are tightly regulated by excessive ROS.^346^ Reviews of the numerous available reports suggest that each hallmark of aging can be linked to the damaging roles of oxidants.^347^ As discussed above, physiological redox signaling is at the core of adaptive homeostasis mechanisms, but aging is always followed by an increasing level of ROS.^348^ Under this condition, an imbalanced redox status prompts ROS‐mediated pathophysiological redox signaling including the classical Nrf2, NF‐κB, and AMPK signaling pathways.^349^, ^350^, ^351^ From the perspective of a single cell, redox imbalance in aging accounts for mitochondrial dysfunction,^352^ protein misfolding and aggregate formation,^353^ abnormal cell membranes and intercellular communication,^354^ and cell death and senescence^355^ by aberrantly modulating multiple signaling pathways, among which mitochondrial dysfunction is strongly associated with aging and aging‐related disorders through ROS control. For example, aging‐induced ROS increased DNA damage and activated DNA‐dependent protein kinase, phosphorylating and counteracting the chaperone function of HSP90‐α for clients that aggravated mitochondrial dysfunction.^356^ Hughes et al. found that the spatial compartmentalization of amino acids by vacuoles was essential for mitochondrial homeostasis in normal cells.^357^ However, aging triggered the breakdown of vacuoles and cysteine accumulation, which remarkably inhibited iron bioavailability and mitochondrial function via ROS. Elevated ROS levels were observed in oocytes during postovulatory aging, which blocked the Sirt1‐FOXO3a‐SOD2 pathway and further increased ROS generation, preventing AKT and ERK1/2 activation and leading to mitochondrial apoptosis.^358^ Wang et al. confirmed that decreased Sirt1 may break mitochondrial biogenesis by increasing PGC‐1‐α acetylation.^359^ On the other hand, mitochondrial dysfunction is also a pivotal inducer of aging. ROS overload was widely found during mitochondrial dysfunction, mediating multiple signaling pathways, such as JNK, to engender cell senescence.^360^ For example, mitochondrial DNA double strand break‐mediated ROS generation accelerated the aging of certain tissues, which can be partially attributed to the activation of cell cycle arrest proteins (p21/p53 pathway).^361^ Thus, there is a positive feedback loop of the aging‐ROS‐mitochondrial dysfunction‐ROS axis, indicating a great potential of modulating ROS levels for attenuating aging (Figure 8).

Dysregulated redox signaling influences lifespan, for which the levels, types, and sites of ROS generation have been identified as important elements.^362^ Different kinds of ROS can have distinct impacts on lifespan. Mounting evidence has indicated the contribution of oxidants to the extension of lifespan. The analysis of genome sequencing of multiple species indicated that the frequency of mitochondria‐encoded cysteine residues negatively correlated with lifespan,^363^ implying that redox biology plays an important part in lifespan and possesses a large therapeutic and commercial potential. Notably, a study conducted by Xiao et al. exploited a new tool to profile the mouse cysteine redox proteome in vivo, which has greatly promoted our knowledge of the underlying mechanisms of redox regulation in aging science.^364^ mitoROS levels seemed to correlate inversely with lifespan, with the overexpression of mitochondrial catalase downregulating H_2_O_2_ levels and extending the lifespan of aged mice.^365^, ^366^ Suhm et al. described how mitochondrial protein translation influenced protein homeostasis and lifespan.^367^ Hypoaccurate translation caused ROS accumulation, limiting lifespan, and proteostasis. However, ROS also seem to function in prolongevity responses through unique pathways. For example, under stress conditions, the intrinsic apoptosis pathway augmented protective mechanisms by sensing mitoROS, which greatly helped control harmful factors.^368^ Independent of DNA damage, cells can also sense mitoROS through epigenetic silencing, which mediates mitochondrial stress‐induced longevity.^369^ AMPK activation strengthened mitochondrial homeostasis and fatty‐acid oxidation, which was essential for the increased lifespan of Caenorhabditis *elegans*.^352^ Another study revealed that ROS generation functioned as an important signal in sustaining mitochondrial homeostasis and extending lifespan in *Drosophila* by inducing reverse electron transport.^370^ Additionally, mitoROS signaling was also found to act on mitohormesis, becoming a significant prolongevity mechanism in response to caloric restriction, hypoxia, body temperature, and physical activity.^371^, ^372^ Intriguingly, a finding by Bazopoulou et al. implied that early‐life H_2_O_2_ exposure benefited later‐life stress resistance and lifespan extension, which may benefit from ROS‐sensitive epigenetic changes in *C. elegans*.^373^

### 4.7 Other diseases

In addition to the above‐mentioned common and serious pathophysiological conditions, other diseases are also intimately correlated with redox dysregulation and oxidative damage. For example, systemic inflammatory response syndrome is an exaggerated host defense response to exogenous factors that generally derive from infectious and noninfectious stimuli, such as sepsis, surgery, and pregnancy, recruiting immune cells to the sites of infection and trauma.^374^ In this process, neutrophils exert essential functions, whose hyperactivation releases a ROS burst and causes subsequent tissue damage. Though the detailed mechanisms still remain to be resolved, the elimination of neutrophils and ROS has been proven to be effective in relieving systemic inflammatory response syndrome.^375^ Fatty liver disease, known as a common liver disorder with excessive accumulation of lipids in the liver, is a redox‐centered disease due to the pivotal function of ROS in hepatic metabolism.^376^, ^377^ In patients with fatty liver diseases, dysregulated lipid metabolism increases the mitochondrial damage and causes ROS overproduction, which facilitates the activation of NACHT, LRR, and PYD domains‐containing protein 3 (NLRP3) inflammasome by damaged hepatic cells, resulting in metabolic inflammation of liver tissues.^378^ Though the underlying mechanisms are still unknown, several studies have suggested that multiple nuclear receptors act as redox sensors and they are promising therapeutic targets in treating fatty liver diseases.^377^ Ischemia‐reperfusion injury (IRI) is accompanied by several major diseases, like heart disease and stroke, which occur under excessive inflammatory responses and OS.^184^ In the reperfusion phase, NO, ONOO^−^, O_2_^−•^ and other oxidants are markedly increased,^379^, ^380^, ^381^ which is followed by elevated levels of OS markers in patients with IRI.^382^ Intriguingly, dysregulation of the circadian rhythm is highly redox‐relevant and contributes to the cardiovascular disease and other pathological conditions.^383^, ^384^ Therefore, several exogenous ROS sources—air pollution and exposure of traffic noise are important risk factors for cardiovascular diseases. The circadian rhythm is tightly controlled by redox status, which is mediated by the redox regulation on cryptochrome 1 (CRY1) and period 2 (PER2) and subsequent activation or repression of circadian locomotor output cycles protein kaput/brain and muscle arnt‐like protein‐1 complex.^383^ OS is a direct cause of acute poisoning, like the chemical herbicide ‐ paraquat. The intake of paraquat leads to rapid and constant accumulation of O_2_^−•^, causing pneumonitis, progressive lung fibrosis, and even death.^385^

## 5 THERAPEUTIC IMPLICATIONS OF REWIRING THE REDOX STATE

Current redox‐based treatment regimens have been widely applied to medical practice. Mainly these are therapeutic pro‐oxidants such as ionizing radiation, oxidant‐generating chemotherapeutics, and photodynamic therapy (PDT). Therapeutic antioxidants are also attracting much interest, some of which have entered clinical trials. However, clinical observations have frequently found only limited clinical benefits and even adverse outcomes for conventional redox‐based therapeutic interventions^386^, ^387^, ^388^, ^389^ (e.g., severe side‐effects and ineffective treatments), which may primarily be attributed to the disruption of ROS signaling and combined metabolic functions. In light of the extensive effects of redox reactions in physiological and pathological conditions, a deeper understanding of the underlying mechanisms of ROS function with a focus on appropriate sources and targets of disease‐relevant ROS will facilitate the development of new‐generation redox and precision medicine. Notably, these concepts are now coming true with some precise therapeutic interventions targeting ROS entering clinical trials.^7^, ^390^

### 5.1 Manipulating redox homeostasis by exogenous pro‐oxidants or antioxidants

Although indiscriminately manipulating redox status to treat diseases has achieved some successes, typically patients suffer from severe side‐effects. On the basis of host defense against invading pathogens that directly release a ROS burst, ionizing radiation has been extensively applied to medical practice, especially for cancer patients. Radiotherapy is one of the earliest paradigms addressing redox medicine and is characterized as the generation of powerful and toxic OH^•^. The adverse effects of radiotherapy on normal tissues vary greatly and include hair loss, anemia, inflammation, epithelial damage, and lung injury.^391^ Although some strategies aimed at precisely identifying the risk of radiation injury, modulating an appropriate radiotherapy dose and improving symptomatic management have been proposed.^391^, ^392^ However, further studies are required in related research fields to further advance treatment potential. Exploiting next‐generation radiotherapy technologies and applying radioprotective drugs could be an option for relieving adverse effects in cancer radiotherapy.^393^, ^394^, ^395^ Traditional oxidant‐generating chemotherapeutics, mostly described as cytotoxic anticancer chemotherapy, target DNA and cause the direct or indirect OS to induce cancer cell death.^396^ Increasing evidence is indicating that the efficacy of conventional anticancer agents not only depends on nonspecific cytotoxic effects but also reactivation of the immune responses of cancer cells, and there is frequent drug resistance and considerable side‐effects (e.g., alopecia, fatigue, nausea, anemia, inflammation, and immunosuppression) which largely restrain their clinical applications.^397^, ^398^ Some studies are trying to resolve the underlying molecular networks behind drug resistance,^398^ but a barrier still remains due to a lack of clinical trials and the intricate tumor heterogeneity.

Photodynamic therapy is another clinically approved and ROS‐elevated treatment regimen, accumulating cytotoxic ROS levels in cancer cells.^399^ PDT utilizes the property of cancer cells preferentially taking up a sensitive photosensitizer (PS), a particular chemical activated by a specific wavelength that releases much ROS (^1^O_2_).^400^ It seems that cancer cells can be targeted and eliminated by PDT, but normal cells can equally absorb PS, leading to the occurrence of unavoidable deleterious side‐effects. In addition,^1^O_2_ only has a short lifetime, which greatly limits its diffusion and narrows the range of action of PDT.^401^ Therefore, although showing some clinical promise, the potential adverse effects and insufficient therapeutic efficacy constrain the further development of PDT.

OS and subsequent disruption of redox signaling are the direct or indirect causes of multiple physiological disorders, which suggest its pharmacological role in choosing therapeutic antioxidants as treatment strategies. However, although many small molecules have been employed as therapeutic antioxidants, clinical observations frequently exhibit poor outcomes.^184^ A clinical trial was conducted to study the effects of several antioxidants (e.g., vitamin C, vitamin E, selenium, I‐carnitine, zinc, folic acid lycopene, and placebo) on semen parameters or DNA integrity among men with infertility. While preclinical studies are promising, the final results suggest that there are no significant improvements in pregnancy or live‐birth rates (NCT02421887).^402^ Another clinical trial was conducted to evaluate the effects of vitamin C and E (initiated between the 9th to 16th weeks) on the risk of pregnancy‐associated hypertension, which also eventually indicated a negative outcome (NCT00135707).^403^ However, systematic antioxidant supplements do help relieve patients with cystic fibrosis, which may be attributed to the specific disease itself or increased systemic antioxidant levels (NCT01018303).^404^ In addition, some antioxidant enzyme mimics also revealed poor prognosis. *N*‐Acetylcysteine (NAC), a well‐established antioxidant agent, has been proven to work in multiple diseases.^405^, ^406^, ^407^, ^408^ However, clinical observation of NAC has been disappointing in many patients including those with hypertrophic cardiomyopathy and type 2 diabetes (NCT01537926, NCT01394510).^409^, ^410^ Therefore, revisiting redox‐modulated treatments from a novel perspective is urgently required to develop new treatments.

### 5.2 Directly targeting ROS sources and redox‐related effectors

Recent ROS‐based drug development emphasizes the importance of modulating the sources and targets of disease‐relevant ROS compared with conventional systematic pro‐oxidant or antioxidant therapy.^411^ Accumulating evidence confirms its feasibility, and some drugs have entered the clinic. NOXs, which are the only enzyme family that are responsible for ROS production, have been shown to be closely related to multiple pathological conditions, such as aging, COPD, diabetes, cardiac dysfunction, and cancer.^250^, ^412^, ^413^, ^414^ Small‐molecule NOX inhibitors based on disease‐related ROS sources have exhibited significant treatment effects. For example, during liver fibrosis, Aoyama and colleagues revealed that the excessive activation of NOX1 in hepatic stellate cells upregulated NOX4 expression, which greatly induced ROS generation and suggested that NOX1/4 were promising therapeutic targets for liver fibrosis.^415^ In vivo experiments confirmed that dual‐targeting treatment with GKT137831, a well‐established NOX1/4 inhibitor,^416^ remarkably attenuated liver fibrosis. Similarly, a dual NOX1/4 inhibitor was also applied to neuroglial cell inflammation and achieved the expected outcomes.^417^ In light of the potent therapeutic effects in preclinical studies, several ongoing or completed clinical trials have been conducted to evaluate the safety and efficacy of GKT137831 in patients with idiopathic pulmonary fibrosis, diabetes, and primary biliary cirrhosis, although the results have not yet been reported (NCT03865927, NCT02010242, NCT03226067). MPO is regarded as an essential promotor in chemoresistant acute myeloid leukemia (AML) cells, preventing increased mitochondrial and cytosolic ROS levels and sensitizing AML cells to cytarabine treatment.^418^ Furthermore, inhibiting MPO activity using the specific inhibitor 4‐aminobenzoic acid hydrazide (ABAH) also dramatically relieved inflammation, increased neurogenesis, and suppressed atherogenesis based on specific mechanisms.^419^, ^420^, ^421^ As well as ABAH, several other MPO inhibitors have also entered clinical studies such as AZD3241 in patients with PD (NCT01603069, NCT01527695) and AZD4831 in patients with heart failure (NCT04232345).

With respect to targets of disease‐relevant ROS, mounting evidence has reported many common and independent downstream molecules in different disease situations. Nrf2, a well‐recognized redox sensor, is activated by elevated ROS levels and evades degradation. Nevertheless, Nrf2 activity is frequently blocked, leading to ROS overload and systematic disorders such as atherogenesis, diabetes, and diabetic nephropathy.^422^, ^423^, ^424^ Nrf2 is capable of stimulating endogenous ROS elimination, which assists the modulation of physiological ROS signaling. Hence, selectively activating Nrf2 is a promising therapeutic strategy for redox‐relevant diseases. Nrf2 activators have been extensively applied in clinical research, among which sulforaphane, synthetic triterpenoids, and dimethyl fumarate are the most successful examples.^184^, ^411^ In preclinical studies, these three compounds were widely reported to function in many disease situations including type 2 diabetes,^425^ epilepsy,^426^ pachyonychia congenita,^427^ cancers,^428^, ^429^, ^430^ autoimmunity,^431^ AD,^432^ and infections.^433^ A large number of clinical trials are currently being carried out in patients with a range of disorders, some of which have undergone preliminary tests and some have even been approved (NCT01335971, NCT03517995, NCT00322140, NCT02683863).

In summary, treatment avenues aimed at inhibiting the sources and targets of disease‐relevant ROS have achieved encouraging preclinical and clinical benefits with excellent safety profiles, which will help facilitate the discovery of more accessible targets and exploitation of more targeted drugs in redox‐relevant diseases. Sophisticated redox control is now gradually replacing conventional systematic redox interventions. Nonetheless, some pivotal questions remain to be resolved. First, identifying the functions of ROS in diseases is the basis, which requires numerous preclinical studies to ensure the reliability. The pathophysiological roles of ROS sources and targets should be complemented under different conditions. Besides, we would better determine the comprehensive landscape of ROS sources and targets in diverse diseases and status of disease progression, achieving which may be conducive to more effective, safe, and precise medical interventions. Various ROS from different sources have been proven to take an important part in disease progression through certain targets and signaling pathways. For monitoring different ROS levels to timely adjust and change therapeutic strategies, a general, accurate, and convenient device needs to be developed. Resolving these problems, the agents directly targeting ROS sources and redox‐related effectors can better serve as next‐generation redox medicine.

## 6 CONCLUSIONS AND PERSPECTIVES

Redox reactions are central to the production of energy, which have given rise to the terms “redox chemistry” and “redox biology.” Generally, redox chemistry involves the transformation of energy and electron transfer processes, which is the basis of human activity. With accumulating knowledge of redox reactions in physiological and pathophysiological conditions, the term “redox biology” has been proposed. Redox homeostasis is mediated by multiple reactive species, in which ROS have been mostly identified as the pivotal mediator. Redox imbalance in favor of oxidant burden frequently emerges, causing various diseases. To overcome this, therapeutic strategies targeting excessive ROS have been developed for many diseases. Unfortunately, while antioxidant therapy has achieved promising effects in many preclinical studies, subsequent clinical trials have often been unsuccessful. Hence, a deep understanding of redox status in pathophysiological conditions is urgently needed to develop new‐generation redox‐relevant agents.

ROS are double‐edged swords. Mounting evidence has indicated a dual role of ROS in human health, which is intricate because of the many elements influencing the final ROS outcome. For example, a moderate level of H_2_O_2_ stimulates multiple physiological signaling pathways through reversible oxidation of specific protein targets (oxidation of sulfur in target proteins), which contributes to the orchestration of multiple cellular biological activities including proliferation, differentiation, and migration. In contrast, excessive H_2_O_2_ generation causes physiological disorders, which result from unspecific oxidation of proteins and irreversible damage to biomacromolecules that are directly linked to growth arrest and cell death. Finding detectable markers of OS in patients with redox‐relevant diseases should be a priority. Balancing the beneficial and toxic effects of ROS will confer great application potential to redox medicine.

In light of the significance of ROS in physiological and pathological conditions, approaches targeting ROS detoxification and production have been widely applied in medical practice. Radiotherapy, a first‐line therapy, can release a ROS burst to kill cancer cells, although this frequently exhibits unwanted and inescapable side‐effects. On the other hand, although ROS detoxification seems to have a huge potential in redox‐relevant diseases, clinical studies have often been associated with poor or even harmful outcomes. Thus, revisiting the role of intervention targeting ROS from a novel perspective will further facilitate the development of redox medicine.

Accumulating reports show that the switch from general to sophisticated redox manipulation on the basis of an understanding of the regulatory mechanisms and sources of disease‐relevant ROS in treating physiological disorders is attracting much interest. This will lead to the discovery of more novel ROS‐related targets and the exploitation of targeted therapies. These methods have prompted new therapeutic strategies on the basis of ROS, some of which are being evaluated in the clinic. In addition, the emerging field of ROS­based nanomedicine also holds great promise for improved clinical applications. The rise of network medicine, integrating phenotypic analysis and intracellular and intercellular connectivity, will link the different omics approaches to mitochondrial function and find more rationale targets on the basis of ROS regulation.

In summary, redox homeostasis is essential for human health, whose imbalance involves nearly all major diseases. Redox regulation as a promising treatment avenue has achieved some success, but the limited efficacy often observed requires the intrinsic mechanisms involved to be further investigated. With new emerging technological advancements in associated fields, we believe that redox regulation will play a more important role in disease management and advancing precision redox medicine.

## CONFLICT OF INTEREST

Canhua Huang is an editorial board member of MedComm. Author Canhua Huang was not involved in the journal's review of, or decisions related to, this manuscript. The other authors have no conflicts of interest to declare.

## ETHICS STATEMENT

Not applicable.

## AUTHOR’S CONTRIBUTIONS

CHH, WZ, and CW conceived the structure of the manuscript. JZ, ZZ, MCL, and LZ drafted initial manuscript. ECN prepared and revised the manuscript. ZZ and JZ prepared the figures. All authors read and approved the final manuscript.

## Data Availability

Not applicable.
